# Network meta-analysis: application and practice using R software

**DOI:** 10.4178/epih.e2019013

**Published:** 2019-04-08

**Authors:** Sung Ryul Shim, Seong-Jang Kim, Jonghoo Lee, Gerta Rücker

**Affiliations:** 1 Department of Preventive Medicine, Korea University College of Medicine, Seoul, Korea; 2 Urological Biomedicine Research Institute, Soonchunhyang University Hospital, Seoul, Korea; 3 Department of Nuclear Medicine, Pusan National University Yangsan Hospital, Pusan National University School of Medicine, Yangsan, Korea; 4 BioMedical Research Institute for Convergence of Biomedical Science and Technology, Pusan National University Yangsan Hospital, Yangsan, Korea; 5 Department of Internal Medicine, Jeju National University Hospital, Jeju National University School of Medicine, Jeju, Korea; 6 Institute of Medical Biometry and Statistics, Faculty of Medicine and Medical Center, University of Freiburg, Freiburg, Germany

**Keywords:** Network meta-analysis, Multiple treatments meta-analysis, Mixed treatment comparison, Consistency, Transitivity, Bayes’ theorem

## Abstract

The objective of this study is to describe the general approaches to network meta-analysis that are available for quantitative data synthesis using R software. We conducted a network meta-analysis using two approaches: Bayesian and frequentist methods. The corresponding R packages were “gemtc” for the Bayesian approach and “netmeta” for the frequentist approach. In estimating a network meta-analysis model using a Bayesian framework, the “rjags” package is a common tool. “rjags” implements Markov chain Monte Carlo simulation with a graphical output. The estimated overall effect sizes, test for heterogeneity, moderator effects, and publication bias were reported using R software. The authors focus on two flexible models, Bayesian and frequentist, to determine overall effect sizes in network meta-analysis. This study focused on the practical methods of network meta-analysis rather than theoretical concepts, making the material easy to understand for Korean researchers who did not major in statistics. The authors hope that this study will help many Korean researchers to perform network meta-analyses and conduct related research more easily with R software.

## INTRODUCTION

Network meta-analysis (NMA), also called multiple treatment meta-analysis, or mixed treatment comparison, aims to synthesize the effect sizes of several studies that evaluate multiple interventions or treatments [1-4].

In the conventional pairwise meta-analysis, the researchers collect studies that evaluate the same treatment, create pairs of the treatment group and control group, and directly calculate the effect size (direct treatment comparison). However, NMA can calculate the effect size between treatment groups through indirect treatment comparison, even if there is no direct comparison study, or if the treatments are different between the treatment groups.

In the present study, the previous meta-analysis studies [1-3] are reviewed using R software. This study focuses on the technical implementation of Bayesian NMA and frequentist NMA using R. Thus, it requires understanding of the direct treatment comparison (which is the basic principle of NMA), indirect treatment comparison through common comparators, and mixed treatment comparison that combines direct and indirect treatment comparisons, as well as prior learning about the assumptions of NMA. These concepts are described in previous studies [1-3].

## STATISTICAL APPROACH OF NETWORK META-ANALYSIS

The NMA methods are largely divided into Bayesian methods and frequentist methods. These two statistical methods have different basic concepts for approaching the statistical model, but produce the same results if the sample size is large.

The Bayesian method calculates the posterior probability that the research hypothesis is true by adding the information given in the present data (likelihood) to previously known information (prior probability or external information). Therefore, it can be said that the Bayesian method is a probabilistic approach, where the probability that the research hypothesis is true can be changed depending on the prior information [1,2].

In contrast, the frequentist method calculates the probability of significance (in general, p-value is 0.05) or the 95% confidence interval (CI) for rejecting or accepting the research hypothesis when the present data is repeated infinitely based on a general statistical theory. Therefore, the frequentist method is unrelated to external information, and the probability that the research hypothesis is true within the present data (likelihood) is already specified, and it only determines whether or not to accept or reject it based on the significance level [1,2].

### Bayesian method

The frequentist method considers the parameters that represent the characteristics of the population as fixed constants and infers them using the likelihood of the given data. However, the Bayesian method expresses the degree of uncertainty with a probability model by applying the probability concept to the parameters.

The most important characteristics of the Bayesian method are as follows.

First, it can use prior information. For example, if the prior information of the parameter of interest exists (from previous research or empirical knowledge of the relevant disease), updated posterior information can be inferred by adding the prior information to the present data. This is much more logical and persuasive than the frequentist assumption that the given data is repeated infinitely.

Second, it is free from the large sample assumption, because the parameters are considered as random variables. For example, the frequentist meta-analysis assumes that the overall effect size follows a normal distribution. In other words, the normality assumption of the normal distribution is satisfactory for a large sample, but most meta-analysis studies have a small number of studies, and the overall effect size may be biased. However, the Bayesian method calculates the posterior information by adding prior information to the likelihood of the given data, and the parameters are probability concepts that can change continuously. Thus, it is free from the effect of a large sample [2,5].

#### Prior and posterior distribution in Bayesian inference

When the parameter of interest is θ, the prior information follows the prior distribution P(θ). When event χ is observed in the present study, the likelihood is P(χ|θ).

Therefore, the posterior distribution function of the updated parameter of interest θ, becomes P(θ|χ) by multiplying the prior distribution by the likelihood function, as follows:

(1) Pθχ=Pθχ*PθPχ

(2) Pθχ∝Pχθ*Pθ

In the conditional probability equation (1), P(χ) is a fixed constant and omitted in general; thus, the equation can be expressed as (2), where ∝ means “proportional”. If the sample size is large, the influence of prior information P(θ) is weak, and a similar result as that of the frequentist method is obtained. However, if the sample size is small and the amount of prior information P(θ) is large, the posterior distribution will produce a different result. Therefore, the Bayesian method generally shows the sensitivity analysis according to the prior distribution.

Prior distributions are largely divided into subjective and objective. The subjective prior distribution can reflect the results of previous studies or empirical knowledge of the disease. In contrast, the objective prior distribution is an informationless prior distribution, which must be objectively quantified and input to the prior distribution.

The prior distribution that is the same as the posterior distribution is called a conjugate prior distribution. The posterior distribution is inferred using the normal distribution if the parameter is average, beta distribution if it is a ratio, or inverse gamma distribution if it is variance.

For non-conjugate prior distribution, it is not easy to integrate the probability of the derived posterior distribution, because it is not generally used in statistical models [5].

#### Markov chain Monte Carlo simulation

For distributions commonly used in statistics, the area under the distribution curve can be simply obtained with an integral formula. However, with the Bayesian method, it is difficult to calculate it if the posterior distribution does not follow a commonly used distribution. In this case, the Markov chain Monte Carlo (MCMC) simulation can be used to calculate it reversely. In this study, we will briefly review the concepts of MCMC as a tool for Bayesian inference.

Markov chain

In a Markov chain, the probability that a random variable will reach a certain state depends only on the previous state.

Next state=current state * transition probability

Therefore, the next state is determined by the likelihood ratio of the current state and the transition probability, which is prior information. In the initial simulation, the value of the next state is significantly different from the current state, but when this calculation is repeated, the difference becomes very small at a certain time, and it reaches a stable steady-state distribution.

To summarize again, the Markov chain uses an algorithm that calculates the probability of the next state knowing the current state and transition probability, and there is no change in the probability after a certain number of repeated calculations.

Monte Carlo simulation

**Figure 1.**
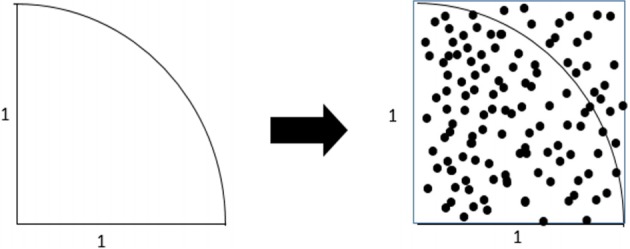
Monte Carlo simulation.

In Figure 1, let us assume that we want to find the area of a 1/4 circle with a radius of 1. This can be calculated easily by 1/4 * πr^2^=1/4*3.142*1^2^, which is 0.7855.

In Monte Carlo simulation, a square around the 1/4 circle is created, and many dots are printed randomly in the square. Then, the desired area can be determined by comparing the number of dots within a distance of 1 from the center of the circle with the total number of dots. For example, if a simulation is performed in which a total of 100 dots is printed, the number of dots within a distance of 1 from the center of the circle will be approximately 78.55. Certainly, the difference between the simulated values and the measured values will decrease as the number of simulations increases.

#### Bayesian hierarchical model

In the fixed effect model, which only considers the within-study variations, the average and variance of the standard normal distribution are input to the prior distribution as follows:

T_i_ ~ N(θ, v_i_), i=1, …, k
θ ~ N(μ_0_, η0^2^)

where T_i_ is the actual observation effect of the *i*th study, and v_i_ is the variance of the *i*th study. Here, θ is the true value of the treatment effect and a common effect size to be inferred by the fixed effect model.

In the random effect model, which considers both within-study and between-study variations, the total average treatment effect of the population (μ) and the between-study variance (τ^2^) are input to the prior distribution. In turn, μ follows a hyperprior distribution, which is a normal distribution with μ_0_ as mean and η_0_^2^ as variance, and τ^2^ follows a hyperprior distribution with p as mean and q as variance. These parameters μ_0_, η_0_^2^, p, and q of the prior distributions μ and τ^2^ are hyperparameters.

T_i_ ~ N(θ, v_i_), i=1, …, k
θ_i_ ~ N(μ, τ^2^)
μ ~ N(μ_0_, η_0_^2^), τ^2^ ~ IG(p, q)

In this way, in the random effect model, the treatments effect of each study are connected by the hyperprior distributions from θ_i_ to θ_k_. Thus, it is no longer an independent model but a hierarchical model [5].

#### Summary of the Bayesian method

**Figure 2.**
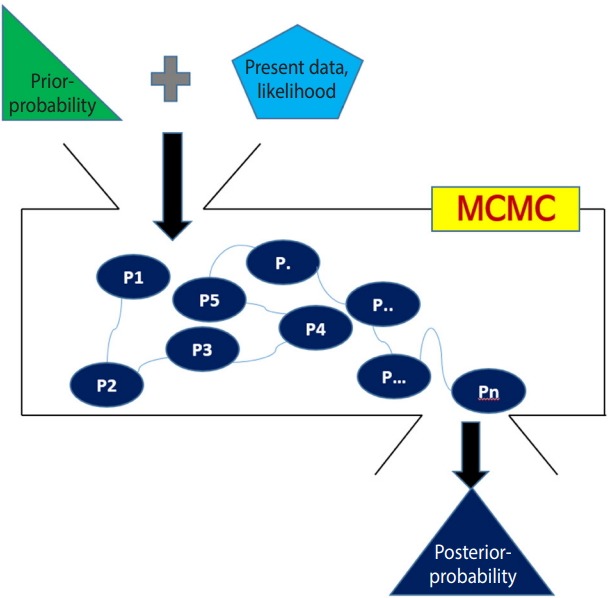
Overall concept of the Bayesian approach using a Markov chain Monte Carlo (MCMC) simulation.

With Figure 2, the Bayesian method can be summarized as follows.

First, a prior distribution (prior probability) is selected. For a conjugate prior distribution, normal distribution, beta distribution, and inverse gamma distribution are generally used.

Second, the likelihood is calculated from the present data and a Bayesian hierarchical model is created – in NMA, the likelihood is mainly expressed as the treatment effect θ.

Third, the prior distribution and likelihood are input to the MCMC simulation, and a distribution that best converges the posterior distribution is set. The probability of stable distribution and the area under the posterior distribution function can be determined through the MCMC simulation.

Lastly, statistical reasoning for the treatment effect is performed with the determined posterior distribution. Therefore, the Bayesian NMA can analyze the posterior distribution even if it is not a standard distribution generally used in statistics.

### Frequentist method

The frequentist method is not related to external information, and the probability that the research hypothesis is true within the present data (likelihood) is already specified (test for a p-value of 0.05 or a 95% CI). Thus, it only determines whether to accept or reject the research hypothesis by the significance level.

The following shows the design by treatment interaction model for inconsistency:

$$y_{\mathrm{di}}^{\mathrm{AJ}}=\delta^{\mathrm{AJ}}+\beta_{\mathrm{di}}^{\mathrm{AJ}}+\omega_{d}^{\mathrm{AJ}}+\varepsilon_{\mathrm{di}}^{\mathrm{AJ}}$$

where A denotes the reference treatment, J denotes the comparative treatment (J=B,C,…), d denotes the study design, and i denotes the *i*th study in the *d*th study design.

The design by treatment interaction model is a frequentist NMA model that considers both heterogeneity between studies and inconsistency between study designs [4].

Here, $y_{\mathrm{di}}^{\mathrm{AJ}}$ represents the effect size of J treatment in contrast to the A reference treatment in the *i*th study of the *d*th study design, considering heterogeneity between studies and inconsistency between study designs. It is called mean difference, log risk-ratio, or log odds ratio (OR). Next, δ^AJ^ represents the treatment contrast as a primary index of interest, and the effect size of J treatment in contrast to the A reference treatment. Then, $\beta_{\mathrm{di}}^{\mathrm{AJ}}$ represents the heterogeneity between studies in the same d study design. It is the same as calculating the interstudy variation τ^2^ with a random effect model in pairwise meta-analysis. Then, $\omega_{d}^{\mathrm{AJ}}$ represents inconsistency. he loop inconsistency is based on Lu & Ades [6], and the design inconsistency was approached through meta-regression analysis of the difference between study designs. Obviously, in the consistency model that satisfies the consistency assumption, $\omega_{d}^{\mathrm{AJ}}$ =0 for all study designs (d) and contrast treatment (J).

$\varepsilon_{\mathrm{di}}^{\mathrm{AJ}}$ is a within-study error. Generally, it is assumed normal distribution.

## BAYESIAN NMA USING R "*gemtc*" PACKAGE

**Figure 3.**
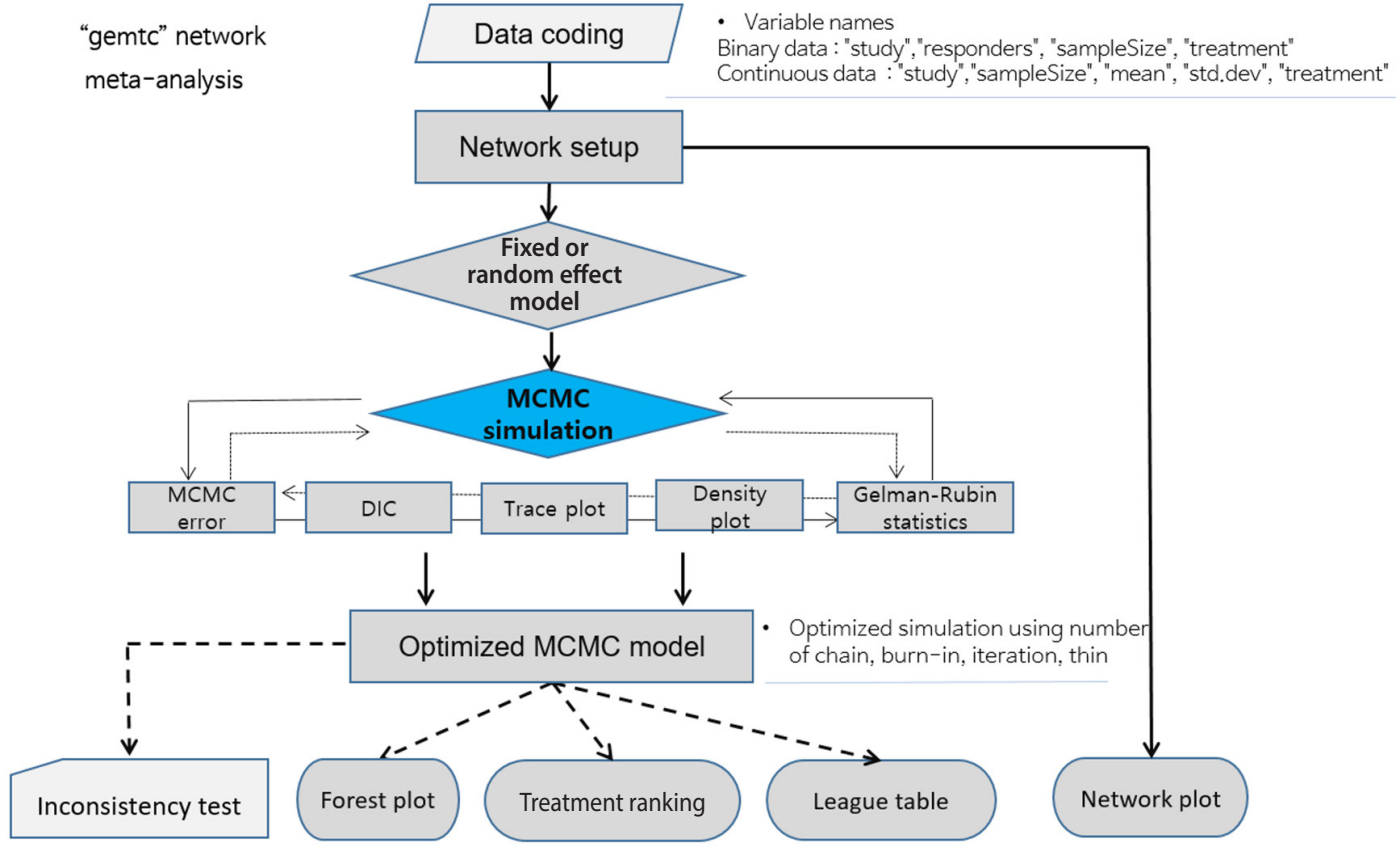
Flow chart of network meta-analysis using the “*gemtc*” R package. MCMC, Markov chain Monte Carlo; DIC, deviance information criterion.

Figure 3 shows the flowchart for using the R package "*gemtc*" for NMA using the Bayesian method. When coding the data first, you must set the variable names in accordance with the relevant function. The process is as follows: network setup -> select a network model (fixed or random) -> select the MCMC convergence optimal model -> statistical reasoning in the final model (Figure 3).

There are two R packages for NMA: “*gemtc*” for Bayesian NMA and “netmeta” for frequentist NMA. Before starting the analysis, you must install the packages with the following commands. For a more detailed explanation, you can refer to the detailed code, data, and references for each package [7].

· install.packages(“netmeta”)

· install.packages(“*gemtc*”)

When running Bayesian NMA, the MCMC simulation is used, and the application required for this is Just Another Gibbs Sampler; Gibbs Sampler is a representative method of MCMC (JAGS). You can download the latest version 4.0 or higher from Google and install it. In addition, you must install the “rjags” package to use JAGS in R as follows:

· install.packages(“rjags”)

We mark R commands with a dot (‘· ’) in front of them, to distinguish them from the main text. When long commands are extended to the next line, there is no dot at the beginning of the next line. Thus, when you enter the command in the R software, you must type them without the dot (‘· ’) in front of them.

### Data coding and loading

First, load the “*gemtc*” package to perform Bayesian NMA.

· library(gemtc)

Next, load the binary example file from the working directory into the memory of R with the following command. Thus, you should save Supplementary Material 1 as “bin_dn.csv” in the specified working folder.

·data_b_bin=read.csv(“bin_dn.csv”, header=TRUE)

This loaded file is saved as “data_b_bin” in the R memory.

The “*gemtc*” package has many sub-functions. Among them, the “mtc.network” function can be run only if the data function name is a specific name. In the binary data, it must be “study,” “responders,” “sampleSize,” or “treatment.” In this example, the variable name is different; thus, you must change the variable name with the “colnames” command as follows:

· colnames(data_b_bin) <- c(“study”, “responders”, “sampleSize”, “treatment”)

“colnames” specifies the variable name (column), and you must sequentially enter the variable names of data_b_bin data.

### Network setup

For a network analysis of the prepared “data_b_bin” data, you must set up the network using the “mtc.network” function as follows:

· network_b_bin <- mtc.network(data.ab=data_b_bin, description=“Bayesian NMA binary data”)

The “mtc.network” function performs network setup with the previously set “data_b_bin” and declares it as “network_b_bin”.

· plot(network_b_bin)

**Figure 4.**
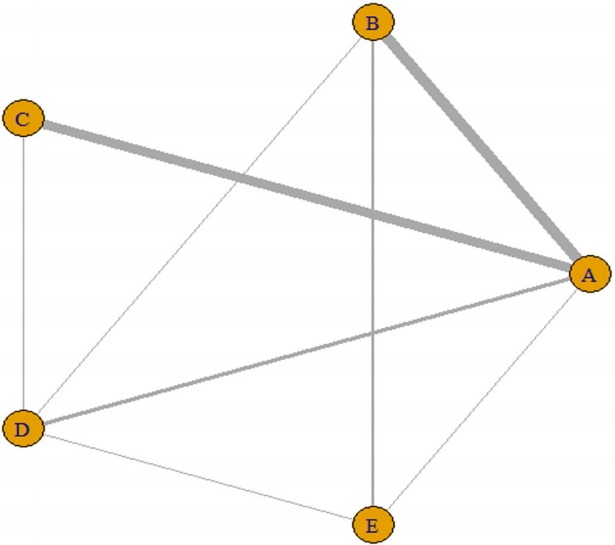
Network plot using the “*gemtc*” package. A: placebo; B: IV (single); C: IV (double); D: topical; E: combination. IV, intravenous injection.

The plot function graphically shows the direct comparison between the treatment groups comprising the network (Figure 4). The thickness of the edge for connecting nodes means the amount of data.

· summary(network_b_bin)

You can see the overall status of network setup. You can also see the number of 2-arm or 3-arm studies and number of responses to individual treatment. Thus, it numerically describes the above network plot.

### Network model

Once the network setup is completed, you must set a network model of fixed effect model or random effect model. Although it is generally recommended to select a random effect model considering the between-study variation, this study will explain with a fixed effect model for convenience.

· model_b_bin_fe <- mtc.model(network_b_bin, linearModel=‘fixed’, n.chain=4)

With the “mtc.model” function, you can load the network setup data “network_b_bin” and set the fixed effect model and random effect model as “model_b_bin_fe”. “n.chain” indicates the number of chains to be performed in the following MCMC simulation.

### Markov chain Monte Carlo (MCMC) simulation and convergence diagnosis

#### Running MCMC simulation

Once the network model is set up, you can perform a MCMC simulation. The overall process is to set and run an appropriate number of simulations, and then check whether the results converge.

First, an example of the fixed effect model will be explained.

· mcmc_b_bin_fe <- mtc.run(model_b_bin_fe, n.adapt=5000, n.iter=10000, thin=20)

In the “mtc.run” function, enter the name of the fixed effect model that has been set. “n.adapt=5000” means to discard no.1-5,000 of the iterations. This is called burn-in, which is to remove a certain part of the beginning of the created random numbers to exclude the effect of the initial values of the algorithm. “n.iter=10000” means to perform 10,000 simulations and “thin=20” means to extract every 20th value.

To summarize the above explanation, the first to 5,000th data are discarded (to reduce the effect of initial values in simulation), simulations are performed 10,000 times with 5,001st to 15,000th values, and every 20th value is extracted (e.g., 5,020, 5,040….).

In the Bayesian analysis, prior distribution considering multichain is input to determine the posterior distribution. The multichain simulation is performed by setting multiple initial values for the prior parameter of prior distribution, that is, the hyperparameter d (e.g., 4 values of -1, 0, 1, and 2). Therefore, because every 20th data are extracted among 10,000 simulations, 500 data points are extracted in each chain.

You can see a more detailed explanation by using the summary command:

· summary(mcmc_b_bin_fe)

#### MCMC simulation and convergence status

To verify if the MCMC simulation converged well, you can check the following items in combination.

MCMC error

A smaller MCMC error indicates a higher accuracy, which means a good convergence. Therefore, a sufficient sample size should be achieved by performing many simulations and the burn-in process to remove the effect of initial values, and the data extraction interval “thin” should be adjusted appropriately.

Deviance information criterion

The deviance information criterion (DIC) is expressed as $DIC=\bar{D}+pD$, where $\bar{D}$ is the sum of residual deviances and *pD* is an estimated value of the parameter. Thus, the DIC considers both the fitness and complexity of the model, and the smaller the DIC is, the better the model.

Trace plot and density plot

If the trace plot (a graph visually showing the simulation result) has no specific pattern and the chains are entangled, it is considered that the convergence is good. The density plot is a posterior distribution (posterior density function) and if the shapes are significantly different for the same number of simulations, it means the data did not converge well.

**Figure 5.**
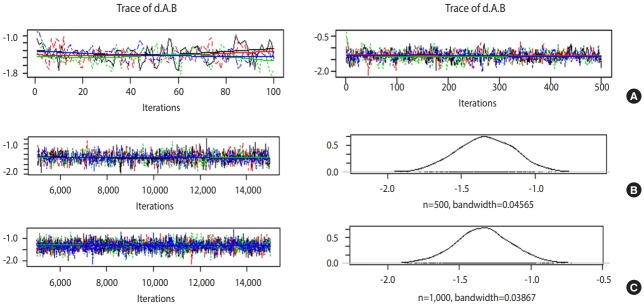
Trace and density plots: (A) iterations=100 (left) vs. 500 (right); (B) iterations=10,000 & thin=20; (C) iterations=10,000 & thin=10.

Figure 5A is the case when 100 and 500 were input as the total number of iterations with no burn-in. In the first 100 iterations (Figure 5A left), the four chains have severe variations and are not even, but at approximately 500th iteration (Figure 5A right), the graph becomes even, with no specific pattern. Therefore, it is desirable to perform at least 500 simulations for each channel. For burn-in to exclude the effect of initial values, 1-100th data points must be discarded without question because they are too uneven, and the number of discarded values must be at least 500. In this example, the values were discarded for up to 5,000 times to minimize the effect of the algorithm.

In Figure 5B, when the simulations are performed 10,000 times with an extraction interval “thin” of 20, 500 data points are extracted from each channel. If 10 is input to thin (Figure 5C), 1,000 values are extracted per channel, which increases the sample size and results in a more even distribution of the trace plot. Furthermore, 1,000 samples of post density function were selected because it looks more similar to the normal distribution.

The finally selected model performed burn-in for 5,000 values and 10,000 simulations, and extracted every 10th data point for 1,000 samples per channel.

· mcmc_b_bin_fe <- mtc.run(model_b_bin_fe, n.adapt=5000, n.iter=10000, thin=10)

Compared to the extraction interval thin of 20, the total sample size increased and the MCMC standard error of the treatment group AB decreased from 0.004 to 0.003, thus increasing the accuracy. However, the DIC decreased from 56.24 to 56.63, which is practically insignificant.

Gelman-Rubin statistics and plot

· gelman.diag(mcmc_b_bin_fe)

· gelman.plot(mcmc_b_bin_fe)

The “gelman.diag” command displays the Gelman-Rubin statistics on the console, and the “gelman.plot” command draws the Gelman-Rubin plot. As the number of simulations increases, it approaches 1, and the variations must be stabilized so that it can be said to have converged well.

##### Selecting the final model for MCMC simulation

For MCMC simulation, a model that converges best should be selected by adjusting the number of chains appropriate for multichain, the number of data for removal of initial effect (burn-in), the number of iterations, and the extraction interval (thin).

For the fixed effect model of this example, 4 chains, 5,000 burnins, 10,000 iterations, and an interval of 10 were selected, to sufficiently remove the effect of initial values, increase the iterations and extraction interval, and minimize the MCMC error and DIC variation with almost no variations and stability of various plots.

However, you should adjust the iterations appropriately because it can take significant time depending on the computer specifications.

Burn-in 5,000, iteration 10,000, thin 10

· mcmc_b_bin_fe <- mtc.run(model_b_bin_fe, n.adapt=5000, n.iter=10000, thin=10)

· plot(mcmc_b_bin_fe)

· summary(mcmc_b_bin_fe)

· gelman.diag(mcmc_b_bin_fe)

· gelman.plot(mcmc_b_bin_fe)

#### Consistency test

Consistency test in the assumptions of NMA is a critical tool that determines the applicability of NMA results.

· nodesplit_b_bin_fe <- mtc.nodesplit(network_b_bin, linearModel=‘fixed’, n.adapt=5000, n.iter=10000, thin=10)

· plot(nodesplit_b_bin_fe)

· plot(summary(nodesplit_b_bin_fe))

The fixed effect model “nodesplit_b_bin_fe” is created for consistency test by entering the network set-up data in the “mtc.nodesplit” function. The MCMC simulation is also performed.

The variations between treatments and the consistency test results of all individual treatments can be easily seen. As a result of the consistency test, the p-value of treatments E versus D was 0.043, indicating inconsistency. However, no statistical significance was observed in all the other treatments. Therefore, it is desirable to set up a random effect model for a more robust analysis.

#### Forest plot

Forest plot allows graphical comparison of the effect sizes by treatment group through NMA.

· forest(relative.effect(mcmc_b_bin_fe, t1=“A”), digits=3)

**Figure 6.**
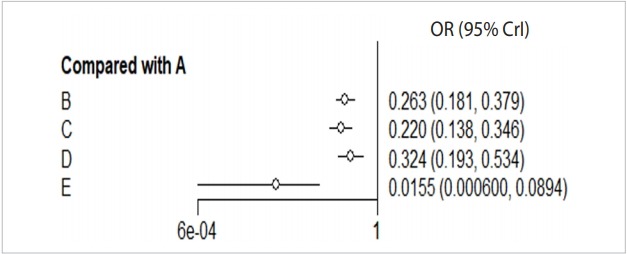
Forest plot_reference A. A: placebo; B: IV (single); C: IV (double); D: topical; E: combination. IV, intravenous; OR, odds ratio; CrI, credible interval.

When you enter the final model through MCMC simulation in the forest function, a forest plot with A reference treatment is created (Figure 6).

The effect size (OR, blood transfusion rate) of every treatment was lower than the placebo, and the 95% credible intervals did not overlap.

In particular, the blood transfusion rate of the combination treatment method (E) was statistically significantly lower compared to those of all the other treatments including single intravenous injection (IV) injection [B, IV(single)], double IV injection [C, IV(double)], and topical application method [D, topical].

#### Treatment ranking

One of the most important functions of NMA is that the comparative advantages of treatments can be determined. In other words, the cumulative probability that the top priority to the lowest priority treatments are selected can be calculated.

· ranks_b_bin_fe <- rank.probability(mcmc_b_bin_fe, preferredDirection=-1)

· print(ranks_b_bin_fe)

Enter the final MCMC model in the “rank.probability” function. Set the “preferredDirection” to ‘-1’ or ‘1’, depending on whether a smaller effect size indicates a better treatment, or vice versa. In this example, it is set to ‘-1’ because an effect size smaller than that of the reference treatment means a better treatment.

As can be seen in the probability table, the best treatment is E (combination) at 99.8%, followed by C (IV double) at 68.2%, B (IV single), D (topical), and A (placebo).

### FREQUENTIST NMA USING R “netmeta” PACKAGE

**Figure 7.**
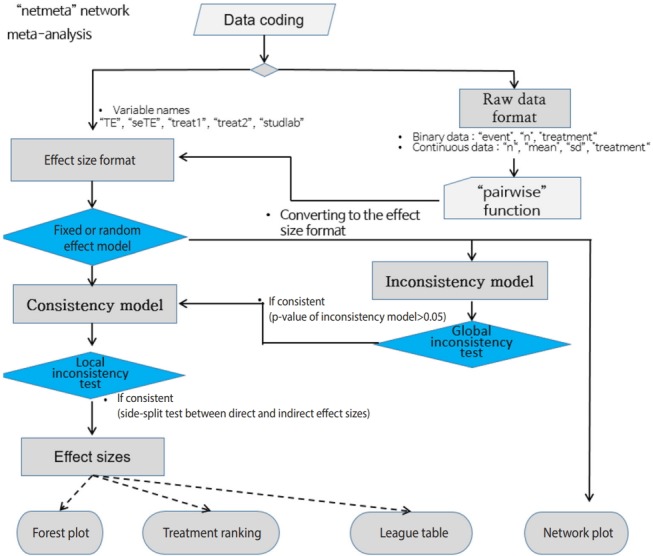
Flow chart of network meta-analysis using the “netmeta” R package.

Figure 7 shows the flowchart for using the R package “netmeta” for NMA using the frequentist method. First, you must change the data format to the effect size data format, and set the variable names in accordance with the relevant function. The process is as follows: effect size data format -> select a network model (fixed or random) -> statistical reasoning in the final model.

#### Data coding and loading Load the “netmeta” package to perform frequentist NMA.

· library(netmeta)

Next, load the binary example file from the working directory into the memory of R with the following command (Supplementary Material 1).

·data_f_bin=read.csv(“bin_dn.csv”, header=TRUE)

The “netmeta” package can be run only if the variable name of the effect size data type is “studlab,” “TE,” “seTE,” “treat1,” or “treat2.” Because the data in this example is raw data, it must be converted to effect size data type, and the variable names must be matched as well.

· data_f_bin <- pairwise(trt, event=d, n=n, studlab=study, data=data_f_bin, sm =“OR” )

Enter the first treatment variable “trt” in the pairwise function, and the other variables are matched: frequency (event=d), sample size (n=n), and study name (studlab=study). Lastly, select whether to use OR or relative risk (RR) data.

#### Network model

If the conversion to the effect size data type has been completed, select the fixed effect model or random effect model for the network model.

· network_f_bin_fe <- netmeta(TE, seTE, treat1, treat2, studlab, data=data_f_bin, sm=“OR”, reference=“A”, comb.fixed=TRUE, comb.random=FALSE)

Set the fixed effect model to network_f_bin_fe by loading effect size (TE), standard error (seTE), treatment 1 (treat1), treatment 2 (treat2), study name (studlab), and data_f_bin in the netmeta function.

##### Network plot

· tname_f_bin <- c(“Placebo”, “IV(single)”, “IV(double)”, “Topical”, “Combination”)

·netgraph(network_f_bin_fe, labels=tname_f_bin)

To enter the treatment name in the network plot, set “tname_f_bin” as the treatment name.

You can enter “network_f_bin_fe” in the “netgraph” function to graphically show a direct comparison between the treatment groups comprising the network. The thickness of the edge for connecting nodes means the amount of data.

##### Network model summary estimates

Once the network model is set up, you can use “summary” to see the overall summary of the model.

· summary(network_f_bin_fe)

The total number of studies is 21, the number of treatments is 5, the number of paired direct comparisons is 25, and the number of study designs is 6 ($\bar{AB},\;\bar{AC},\;\bar{AD},\;\bar{AE},\;\bar{BDE},\;and\;\bar{ABE}$).

Furthermore, the reference variable is A and the effect size is OR.

In this example, the fixed and random effect models have the same effect size because the between-study variance (tau) is zero.

#### Consistency test

Consistency test in the assumptions of NMA is a critical tool that determines the applicability of NMA results.

##### Global approach

This approach calculates the regression coefficient of the inconsistency model for each study design and then tests the linearity of the regression coefficients for all models by using the Wald test. The consistency test was performed for all models in the same way as for the STATA NMA model [4].

· decomp.design(network_f_bin_re)

As a result of the consistency test for every model, the p-value was 0.9942. As this supports consistency, which is the null hypothesis, this network model is acceptable.

##### Local approach

· print(netsplit(network_f_bin_re), digits=3)

Enter the network model in the “netsplit” function and perform a consistency test for each treatment.

In all comparisons of treatments, the p-value was statistically insignificant, showing no inconsistency. Therefore, the consistency model is supported once again.

#### Forest plot

A forest plot allows a graphical comparison of the effect sizes by treatment group through NMA.

· forest(network_f_bin_fe, ref=“A”, digits=3, xlab=“Odds Ratio”)

Enter the set network model and “A” for reference treatment in the forest function.

The effect size (OR, blood transfusion rate) of every treatment was lower than that of the placebo, and the 95% CIs did not overlap.

In particular, the transfusion rate (OR, 0.033; 95% CI, 0.006 to 0.175) of the combination treatment (E) was statistically significantly lower than those of all the other treatments including single IV injection [B, IV (single)] (OR, 0.273; 95% CI, 0.186, to 0.399), double IV injection [C, IV (double)] (OR, 0.229; 95% CI, 0.146, 0.360), and the topical application method [D, topical] (OR, 0.329; 95% CI, 0.197 to 0.550).

#### Treatment ranking

One of the most important functions of NMA is that the comparative advantages of treatments can be determined. In other words, the cumulative probability that the top priority to the lowest priority treatments are selected can be calculated.

· ranks_f_bin_fe <- netrank(network_f_bin_fe, small.values=”good”)

· print(ranks_f_bin_fe, sort=FALSE)

Enter the network model in the “netrank” function. Enter “good” in “small.values” if a smaller effect size indicates a better treatment, or “bad” otherwise.

As can be seen in the probability table, the best treatment is E (combination) at 99.38%, followed by C (IV double) at 65.43%, B (IV single), D (topical), and A (placebo).

## COMPARISON OF NMA RESULTS: BAYESIAN VS. FREQUENTIST METHOD AND R VS. STATA SOFTWARE

Table 1 outlines the Bayesian and frequentist NMA. The NMA used in STATA is a design by treatment interaction model based on regression analysis, which considers both heterogeneity between studies and inconsistence between study designs [4]. However, the R “netmeta” package uses an electrical network model, which changed it slightly [8,9].

The Bayesian method also shows similar values between the fixed and random effect models, which is an almost identical result to that of the frequentist method.

## CONCLUSION

This paper presented as little statistical theory as possible and focused instead on the actual performance of meta-analysis methods, so that researchers who have not majored in statistics could easily understand it. In other words, this study aimed to provide researchers from different fields an overview of how to adequately use already developed statistical methods in their field of study and interpret the results.

This study compared the Bayesian network meta-analysis using the “*gemtc*” package and the frequentist network meta-analysis using the “netmeta” package. We found that these two methods produced the same results. Refer to the references for detailed descriptions for continuous data, besides the binary data presented in the examples in this study [2].

We hope that this study will help researchers to perform metaanalysis and conduct related research more easily.

## Figures and Tables

**Table 1.** Comparison effect sizes between frequentist and Bayesian method in network meta-analysis

Data type	Treatment	Frequentist approach			Bayesian approach^1^	
		STATA^2^	R "nemeta" package		R "*gemtc*" package	
			Fixed	Random	Fixed	Random
Binary	Placebo	1.000 (reference)	1.000 (reference)	1.000 (reference)	1.000 (reference)	1.000 (reference)
	IV (single)	0.273 (0.186, 0.399)	0.273 (0.186, 0.399)	0.273 (0.186, 0.399)	0.263 (0.181, 0.379)	0.264 (0.173, 0.399)
	IV (double)	0.229 (0.146, 0.360)	0.229 (0.146, 0.360)	0.229 (0.146, 0.360)	0.220 (0.138, 0.346)	0.220 (0.138, 0.357)
	Topical	0.329 (0.197, 0.550)	0.329 (0.197, 0.550)	0.329 (0.197, 0.550)	0.324 (0.193, 0.534)	0.322 (0.180, 0.551)
	Combination	0.033 (0.006, 0.175)	0.033 (0.006, 0.175)	0.033 (0.006, 0.175)	0.015 (0.001, 0.089)	0.014 (0.000, 0.083)

Values are presented as odds ratio (95% confidence interval). IV, intravenous injection.

1 Effect size (95% credible interval).

2 Design-by-treatment interaction model.

## SUPPLEMENTARY MATERIALS

Korean version is available at http://www.e-epih.org/.
