# Supplementary material for: Network meta-analysis: application and practice using R software
Source: Epidemiol Health. 2019 Apr 8;41:e2019013. doi: 10.4178/epih.e2019013 (PMC6635665; doi:10.4178/epih.e2019013)
Supplement: Supplementary file 1 [file epih-41-e2019013-supplementary.pdf]

Supplementary Material 1. R 네트워크 메타분석 코드

#예제자료는 bin_dn (Table S1) 임.

▶ 베이지안 방법 “gemtc” 패키지_이분형자료

```
#gemtc 패키지 로딩
library(gemtc)

#데이터 불러오기
data_b_bin = read.csv("bin_dn.csv", header=T)
#R_gemtc; study, treatment, responders, sampleSize 변수명 일치해야한다. 만약 아니라면
#변수이름을 네트워크 셋업시 사용할 mtc.network 함수에서 규정한대로 바꾸어 준다.
colnames(data_b_bin)<-c("study","responders", "sampleSize", "treatment")

#치료 id 생성 (mtc.network함수에서 규정한 변수명이라면 생략가능하다)
treatments_bin <- read.table(textConnection('
                                    id description
                                    A "Treatment A"
                                    B "Treatment B"
                                    C "Treatment C"
                                    D "Treatment D"
                                    E "Treatment E"
                                    '), header=TRUE)
#네트워크 셋업
network_b_bin <-
mtc.network(data.ab=data_b_bin,treatments=treatments_bin,description="Bayesian NMA
binary data") #treatments 인자는 제외하여도 된다.
plot(network_b_bin) #network evidence map
summary(network_b_bin)
print(network_b_bin)

#네트워크 모델 설정
# Fixed effect model
model_b_bin_fe <- mtc.model(network_b_bin, linearModel='fixed', n.chain=4)

###MCMC simulation###
### burn-in 5000, iteration 10000, thin 20 ########
mcmc_b_bin_fe <- mtc.run(model_b_bin_fe, n.adapt=5000, n.iter=10000, thin=20)
plot(mcmc_b_bin_fe)
gelman.diag(mcmc_b_bin_fe)
gelman.plot(mcmc_b_bin_fe)
summary(mcmc_b_bin_fe)
### burn-in 5000, iteration 10000, thin 10 ########
mcmc_b_bin_fe <- mtc.run(model_b_bin_fe, n.adapt=5000, n.iter=10000, thin=10)
plot(mcmc_b_bin_fe)
gelman.diag(mcmc_b_bin_fe)
gelman.plot(mcmc_b_bin_fe)
summary(mcmc_b_bin_fe)
# 결과 보기
```

```
summary(mcmc_b_bin_fe)
forest(mcmc_b_bin_fe)
forest(relative.effect(mcmc_b_bin_fe, t1="A"), digits=3)

### Random effect model
model_b_bin_re <- mtc.model(network_b_bin, linearModel='random', n.chain=4)

###MCMC simulation###
### burn-in 5000, iteration 10000, thin 20 ########
mcmc_b_bin_re <- mtc.run(model_b_bin_re, n.adapt=5000, n.iter=10000, thin=20)
plot(mcmc_b_bin_re)
gelman.diag(mcmc_b_bin_re)
gelman.plot(mcmc_b_bin_re)
summary(mcmc_b_bin_re)
### burn-in 5000, iteration 10000, thin 5 ########
mcmc_b_bin_re <- mtc.run(model_b_bin_re, n.adapt=5000, n.iter=10000, thin=5)
plot(mcmc_b_bin_re)
gelman.diag(mcmc_b_bin_re)
gelman.plot(mcmc_b_bin_re)
summary(mcmc_b_bin_re)
# 결과 보기
summary(mcmc_b_bin_re)
forest(mcmc_b_bin_re)
forest(relative.effect(mcmc_b_bin_re, t1="A"), digits=3)

###Inconsistency 가정 확인: node-splitting을 통해서########
#mtc.nodesplit에 네트워크 셋업을 넣는다.
#fixed moel
nodesplit_b_bin_fe <- mtc.nodesplit(network_b_bin, linearModel='fixed', n.adapt=5000,
n.iter=10000, thin=10)
#이건 하지말자 너무 많이 나옴 plot(nodesplit_b_bin_fe)
plot(summary(nodesplit_b_bin_fe))
#random moel
nodesplit_b_bin_re <- mtc.nodesplit(network_b_bin, linearModel='random', n.adapt=5000,
n.iter=10000, thin=5)
#이건 하지말자 너무 많이 나옴 plot(nodesplit_b_bin_re)
plot(summary(nodesplit_b_bin_re))

###치료법간 비교우위 선정###
#rank.probability에 mcmc 시뮬레이션 결과값을 넣는다.
#확률이 높은쪽이 선호되면 preferredDirection을 1을 넣고 낮은쪽이 선호되면 -1을 넣
는다.
#fixed moel
ranks_b_bin_fe <- rank.probability(mcmc_b_bin_fe, preferredDirection = -1)
print(ranks_b_bin_fe)
plot(ranks_b_bin_fe, beside=TRUE)
#random moel
ranks_b_bin_re <- rank.probability(mcmc_b_bin_re, preferredDirection = -1)
print(ranks_b_bin_re)
plot(ranks_b_bin_re, beside=TRUE)
```

▶ 빈도주의 방법 “netmeta” 패키지_이분형자료

```
#netmeta 패키지 로딩
```

```
library(netmeta)

#데이터 불러오기
data_f_bin = read.csv("bin_dn.csv", header=T)

####raw data가 event n이라면 TE seTE형태로 바꾼다.###
#TE seTE형태라면 그냥 바로 netmeta실시하면된다.
#Binary data; dn raw data를 TE seTE로 변환하기; pairwise 명령어
#변수이름을 임의대로 바꿀수있다.
colnames(data_f_bin)<-c("studlab","event", "n", "treatment")
#pairwise로 효과크기 만들때 OR(로그변환된)로 할것인지 RR(로그변환된) 할 것인지가 중요함.
data_f_bin <- pairwise(treatment, event = event, n = n,
                       studlab = studlab, data = data_f_bin, sm ="OR" )
##변수이름을 바꾸지 않았다면 study, d, n, trt 일때
#data_f_bin <- pairwise(trt, event = d, n = n, studlab = study, data = data_f_bin, sm ="OR" )
#data_f_bin를 가지고 기존의 netmeta를 돌리면된다.
###

#네트워크 플롯에서 변수명을 직접 치료명으로 표기할때 사용한다.
tname_f_bin <- c("Placebo", "IV(single)", "IV(double)", "Topical", "Combination")

#네트워크 모델 설정

##Fixed effect model##
network_f_bin_fe <- netmeta(TE, seTE, treat1, treat2,
                            studlab, data=data_f_bin, sm="OR", reference="A", comb.fixed=TRUE, comb.random=FALSE)
netgraph(network_f_bin_fe, labels=tname_f_bin) #네트워크 plot 생성
forest(network_f_bin_fe, ref = "A", digits=3,  xlab = "Odds Raio")
summary(network_f_bin_fe)
print(network_f_bin_fe)

##일관성 검정##
#inconsistency test(global approach) design-by-treatment interaction random effects model
decomp.design(network_f_bin_fe)
#inconsistency test(local approach) side split (direct vs indirect comparision)
print(netsplit(network_f_bin_fe), digits=3)

##치료간 비교우위 선정; rank test##
ranks_f_bin_fe <- netrank(network_f_bin_fe, small.values="good")
print(ranks_f_bin_fe, sort=FALSE)

##Random effect model##
network_f_bin_re <- netmeta(TE, seTE, treat1, treat2,
                            studlab, data=data_f_bin, sm="OR", reference="A", comb.fixed=FALSE, comb.random=TRUE)
netgraph(network_f_bin_re, labels=tname_f_bin) #네트워크 plot 생성
forest(network_f_bin_re, ref = "A", digits=3,  xlab = "Odds Raio")
summary(network_f_bin_re)
print(network_f_bin_re)

##일관성 검정##
```

```
#inconsistency test(global approach) design-by-treatment interaction random effects model
decomp.design(network_f_bin_re)
#inconsistency test(local approach) side split (direct vs indirect comparision)
print(netsplit(network_f_bin_re), digits=3)

##치료간 비교우위 선정; rank test##
ranks_f_bin_re <- netrank(network_f_bin_re, small.values="good")
print(ranks_f_bin_re, sort=FALSE)
```

Table S1. Binary sample data for network meta-analysis

study	d	n	trt
Alshryda 2013	10	80	D
Alshryda 2013	26	81	A
Barrachina 2016	4	36	C
Barrachina 2016	14	37	A
Benoni 2000	9	20	C
Benoni 2000	15	19	A
Benoni 2001	4	18	B
Benoni 2001	8	20	A
Claeys 2007	1	20	B
Claeys 2007	6	20	A
Fraval 2017	1	50	C
Fraval 2017	6	51	A
Husted 2003	2	20	C
Husted 2003	7	20	A
Hsu 2015	2	30	C
Hsu 2015	9	30	A
Johansson 2005	8	47	B
Johansson 2005	23	53	A
Kazemi 2010	7	32	B
Kazemi 2010	15	32	A
Lee 2013	9	34	C
Lee 2013	20	34	A
Lemay 2004	6	20	C
Lemay 2004	13	19	A
Martin 2014	3	25	D
Martin 2014	5	25	A
Niskanen 2005	5	19	C
Niskanen 2005	8	20	A
North 2016	8	70	C
North 2016	12	69	D
Rajesparan 2009	3	36	B
Rajesparan 2009	10	37	A
Wang 2016	9	81	B
Wang 2016	10	38	A
Wei 2014	6	101	B
Wei 2014	26	100	A
Xie 2016	3	70	B
Xie 2016	4	70	D
Xie 2016	0	70	E
Yi 2016	8	50	B
Yi 2016	1	50	E
Yi 2016	19	50	A
Yue 2014	3	52	D
Yue 2014	11	49	A

d, events; n, total sample sizes; trt, treatment. A, Placebo; B, IV(single); C, IV(double); D, Topical; E, Combination. [1,2].
