# Supplementary material for: Network meta-analysis: application and practice using R software
Source: Epidemiol Health. 2019 Apr 8;41:e2019013. doi: 10.4178/epih.e2019013 (PMC6635665; doi:10.4178/epih.e2019013)
Supplement: Supplementary file 2 [file epih-41-e2019013-supplementary1.pdf]

Volume: 41, Article ID: e2019013

https://doi.org/10.4178/epih.e2019013

<방법론>

네트워크 메타분석: R을 활용한 적용과 실제

Network Meta-analysis: Application and Practice using R software

심 성 률[1,2], 김 성 장[3,4,5], 이 종 후[6], Gerta Rücker[7]

[1]고려대학교 의과대학 예방의학교실, [2]순천향대학교 서울병원 비뇨의과학연구소, [3]부산대학교 의과대학 핵의학과, [4]양산부산대학교병원, 의생명융합연구소, [5]양산부산대학교병원, 핵의학과, [6]제주대학교 의과대학 내과, [7]Institute of Medical Biometry and Statistics, Faculty of Medicine and Medical Center – University of Freiburg

Sung Ryul Shim, MPH, PhD[1,2], Seong-Jang Kim MD,PhD[3,4,5], Jonghoo Lee, MD, PhD[6], Gerta Rücker[7]

[1]Department of Preventive Medicine, Korea University College of Medicine, Seoul, Korea, [2]Urological Biomedicine Research Institute, Soonchunhyang University Hospital, Seoul, Korea, [3]Department of Nuclear Medicine, College of Medicine, Pusan National University, Yangsan, Korea, [4]BioMedical Research Institute for Convergence of Biomedical Science and Technology, Pusan National University Yangsan Hospital, Yangsan, Korea, [5]Department of Nuclear Medicine, Pusan National University Yangsan Hospital, Yangsan, Korea, [6]Department of Internal Medicine, Jeju National University Hospital, Jeju National University School of Medicine, Jeju, Korea, [7]Institute of Medical Biometry and Statistics, Faculty of Medicine and Medical Center - University of Freiburg

교신저자: 심성률

sungryul.shim@gmail.com

서울시 성북구 안암로 145

Volume: 41, Article ID: e2019013

https://doi.org/10.4178/epih.e2019013

Abstract

The objective of this study was to describe general approaches of network meta-analysis that is available for quantitative synthesis of data using R software.

We conducted a network meta-analysis using two types of concepts: Bayesian and frequentist method.

The packages of R software were gemtc for Bayesian approach and netmeta for frequentist approach.

In estimating a network meta-analysis models in a Bayesian framework, rjags package is one of conformance fitting tools. The package of rjags implemented Markov Chain Monte Carlo simulation with graphical outcomes. The estimated overall effect sizes, test for heterogeneity and moderator effect, and the publication bias were reported using R software. Especially authors stressed two flexible modellings of Bayesian and frequentist for overall effect sizes in network meta-analysis. This study focused on the practical methods of network meta-analysis rather than theoretical concepts for Korean researchers who were non-majored in statistics. Through this study, authors hope that many Korean researchers will use R software to perform a network meta-analysis more easily and that related research will be activated.

Keywords: Network Meta-analysis, Multiple treatments meta-analysis, Mixed treatment comparison, Consistency, Transitivity, gemtc, netmeta, Bayesian theorem.

서론

네트워크 메타분석(NMA, network meta-analysis)은 multiple treatment meta-analysis 또는 mixed treatment comparison이라고도 하며 다수의 치료(intervention or treatment)를 가진 여러 연구들의 효과크기를 종합하는 것이다 [1-4].
기존의 일반적인 메타분석(pairwise meta-analysis)은 동일한 치료를 실시한 연구들을 모아 치료군과 비교군으로 두 군의 짝을 만든 후 효과크기를 직접 계산하였다(direct treatment comparison). 그러나 네트워크 메타분석은 치료그룹별 직접비교 연구가 없거나 치료가 서로 다르다고 하더라도 치료그룹 간의 효과크기를 간접비교(indirect treatment comparison) 방법을 이용하여 계산할 수 있다.

본 연구는 저자가 수행했던 메타분석 선행연구들을 [1-3] R 소프트웨어를 이용해서 다시 풀어낸 것이다. 또한 본 연구는 R을 이용한 베이지안 네트워크 메타분석과 빈도주의 네트워크 메타분석의 기술적인 실행에 초점을 맞추고 있기 때문에 네트워크 메타분석의 기본원리인 직접비교, 공통 비교인자(common comparator)를 통한 간접비교, 그리고 직접비교와 간접비교를 종합한 혼합비교(mixed treatment comparison)에 대한 이해와 더불어 네트워크 메타분석에 대한 가정(assumption)들에 대한 선행학습이 필수적이다. 따라서 국문으로 되어진 선행연구[1-3]를 통하여 충분한 개념 정립이 수반되어야 한다.

1. 네트워크 메타분석의 통계적 접근

네트워크 메타분석을 실행하는 방법은 베이지안(Bayesian) 방법과 빈도주의(Frequentist) 방법으로 나누어진다. 두 통계 방법은 통계모형에 접근하는 근본적인

개념이 상이하지만 표본수가 많을 경우 동일한 결과를 도출한다.

베이지안 방법은 사전에 알려진 정보(prior probability 또는 external information)를 바탕으로 현재의 자료에서 주어진 정보를(present data, likelihood) 더하여 연구가설이 참(true) 일 사후확률(posterior probability)을 산출하는 것이다. 따라서 베이지안 방법은 사전정보에 따라 연구가설이 참 일 확률이 변할 수 있는 확률적인 접근이라고 할 수 있다 [1,2].

반면에 빈도주의 방법은 일반적인 통계이론에 근거하여 현재의 주어진 자료가 (present data) 무한히 반복되었을 때 연구가설이 기각되거나 받아들여질 유의확률 (일반적으로 type 1 error, p-value 0.05) 또는 신뢰구간을 검정함으로써 산출하는 것이다. 따라서 빈도주의 방법은 외적정보와는 무관하며 주어진 자료(present data, likelihood)내에서 연구가설이 참 일 확률은 이미 특정되어 있어서 단지 이것이 받아들여지거나 기각될지를 유의수준으로 판단한다 [1,2].

1.1. 베이지안 방법

빈도주의적 방법에서는 모집단의 특성을 나타내는 모수(parameter)를 고정된 상수로 보고 주어진 자료의 우도(likelihood)를 이용하여 이를 추론한다. 그러나 베이지안 방법에서는 모수는 불확실성을 지니기 때문에 모수에 확률 개념을 적용하여 불확실성의 정도를 확률모형으로 표현한다.

따라서 베이지안의 여러 장점 중 가장 두드러지는 특징은 다음과 같다.

첫째, 사전정보를 활용할 수 있다는 것이다. 예를 들면 만일 관심 모수의 사전정보

https://doi.org/10.4178/epih.e2019013

가 선행연구 또는 해당 질환의 경험적 지식 등으로 존재할 경우 이를 현재의 자료에 넣어서 보다 업데이트 된 사후정보를 추론할 수 있다. 이것은 주어진 자료가 무한이 반복된다는 빈도주의적 가정에 비해 훨씬 논리적이며 설득력이 있다.

둘째, 모수를 확률변수로 보기 때문에 대표본 가정에서 자유롭다. 예를 들면 빈도주의 메타분석에서 통합된 효과크기는(overall effect size) 정규분포를 따른다고 가정한다. 즉, 정규분포의 정규성 가정은 대표본일 경우 만족스러우나 대부분의 메타분석 연구들은 연구의 수가 적어 통합된 효과크기에 편향이 생길 수 있다. 그러나 베이지안 방법에서는 주어진 자료의 우도에 더해지는 사전정보를 종합하여 사후정보를 계산하기 때문에 모수는 계속해서 변할 수 있는 확률개념이라서 대표본의 영향에서 자유롭다 [2,5].

1) 베이지안 추론에서의 사전분포와 사후분포

관심모수를 θ라고 할 때, 사전정보는 사전분포 P(θ)를 따르며 현재의 연구에서 자료(χ)가 주어졌을 때 우도(likelihood)는 P(χ|θ)이다.
따라서 업데이트 되는 관심모수 θ의 사후분포함수는 사전분포와 우도함수를 곱하여 P(θ|χ)가 된다.

$$P(\theta|\chi) = \frac{P(\chi|\theta) * P(\theta)}{P(\chi)} \quad \text{------ ①}$$

$$P(\theta|\chi) \propto P(\chi|\theta) * P(\theta) \quad \text{------ ②}$$

조건부 확률 공식 ①에서 P(χ)는 고정된 상수이기 때문에 일반적으로 생략하여 공식 ②로 표현한다. ∝는 비례한다는 의미이며, 주어진 표본의 수가 많다면 사전정보

Volume: 41, Article ID: e2019013

https://doi.org/10.4178/epih.e2019013

[P(θ)]의 영향력이 약해져서 빈도주의적 방법과 유사한 결과를 나타낸다. 그러나 반대로 표본의 수가 적고 사전정보[P(θ)]의 양이 크다면 사후분포는 상이한 결과를 나타낼 것이다. 따라서 베이지안 방법에서는 사전분포에 따른 민감도 분석을 보여주는 것이 일반적이다.

사전분포는 크게 주관적(subjective) 사전분포와 객관적(objective) 사전분포로 나뉜다.
주관적 사전분포는 선행연구결과나 해당질환의 경험지식 등이 있을 경우 이를 반영하여 정할 수 있다. 이와는 반대로 객관적 사전분포는 무정보적인 사전분포로서 이를 객관적으로 계량화해서 사전분포로 넣어주어야 한다.
이때 사후분포와 동일한 분포를 지니는 사전분포를 공액사전분포(conjugate prior)라고 하며 모수가 평균인 경우 정규분포, 비율인 경우 베타분포, 분산인 경우 역감마분포 등을 사용하여 사후분포를 추론한다.

비공액사전분포의 경우 일반적으로 통계모형에서 활용하는 분포가 아니기 때문에 도출되는 사후분포의 확률을 적분해내기 쉽지 않다 [5].

2) 마르코프연쇄 몬테카를로(Markov Chain Monte Carlo, MCMC) 시뮬레이션

일반적으로 통계학에서 많이 쓰이는 분포들을 사용한다면 분포곡선아래의 면적을 계산해내는 것은 적분공식을 활용하면 간단히 구할 수 있다. 그러나 베이지안 방법에서는 사후분포가 일반적으로 사용되는 분포를 따르지 않는다면 계산이 힘들어진다. 이를 경우 MCMC 시뮬레이션을 활용하여 역으로 이를 계산해 낼 수 있으며 본

연구에서는 베이지안 추론을 위한 도구로서 MCMC에 대해서 간략한 개념을 알아보도록 하자.

(1) 마르코프 연쇄(Markov chain)

마르코프 연쇄에서는 확률변수(random variable)가 어떤 상태(state)에 도달할 확률이 오직 바로 이전 시점의 상태(state)에 달려 있다.

다음상태(next state) = 현재상태(current state) * 전이확률(transition probability)

따라서 다음상태는 현재상태의 우도비와 사전정보인 전이확률에 따라서 결정된다. 초기 시뮬레이션에서는 다음상태의 값이 현재상태에 비해 차이가 나지만 이러한 계산을 계속해 나가면 어느 횟수에서는 거의 차이가 없으며 상태가 일정해지는 stable distribution에 도달하게 된다.

다시한번 정리하자면 마르코프 연쇄는 일종의 알고리즘으로서 현재상태와 전이확률로서 다음상태의 확률을 계산해내며 일정 횟수가 반복되면 더 이상 확률의 변화는 없다는 것을 알 수 있다.

(2) 몬테카를로(Monte carlo) 시뮬레이션

Figure 1에서 반지름이 1인 1/4 크기 원의 넓이를 구한다고 가정하자. 반지름이 1인 1/4 크기 원의 넓이는 1/4 * πr2 = 1/4*3.142*1^2 으로서 0.7855 를 쉽게 계산해 낼 수 있다.

이것을 몬테카를로 시뮬레이션에서는 우선 1/4 크기의 원을 둘러싸는 정사각형을

Volume: 41, Article ID: e2019013

https://doi.org/10.4178/epih.e2019013

만든 다음 사각형안에 무작위로 많은 점을 찍는다. 그런 다음 원의 중심에서 거리가 1이내에 찍힌 점의 개수와 전체 점의 개수를 비교하면 원하는 넓이를 구할 수 있는 것이다. 예를 들어 전체 100개의 점을 찍는 시뮬레이션을 실시했다고 하면 원의 중심에서 1이내에 찍힌 점의 개수는 약 78.55개에 달할 것이다. 당연히 시뮬레이션의 횟수가 높아질 수록 실측치와의 오차는 줄어들게 된다.

3) 베이지안의 계층적 모형

연구내 변량만 고려하는 Fixed effect model은 사전분포에 표준 정규분포의 평균(μ)과 분산(η^2)을 넣어준다.

$$T_i \sim N(\theta, v_i),\ i = 1, \ldots, k$$
$$\theta \sim N(\mu_0,\ \eta_0^2)$$

T_i는 i 번째 연구의 실제 관찰효과이며, v_i 는 i번째 연구의 분산이다. θ는 치료효과의 참값으로 Fixed effect model에서 추정하고자 하는 하나의 공통된 효과크기이다.

연구내 변량과 연구간 변량을 동시에 고려하는 Random effect model은 사전분포에 모집단의 전체 평균치료효과 평균(μ)과 연구간 분산(τ^2)을 넣어주는데 μ는 다시 평균 μ_0, 분산 η_0^2로하는 정규분포인 초사전 분포(hyperprior distribution)를 따르며, τ2 역시 평균 p, 분산 q로하는 역감마 분포인 초사전 분포를 따른다. 이처럼 사전분포 μ와 τ2 가 가지는 모수 μ_0, η_0^2, p, q는 초모수(hyperparameter)이다.

$$T_i \sim N(\theta, v_i),\ i = 1, \ldots, k$$
$$\theta_i \sim N(\mu, \tau^2)$$
$$\mu \sim N(\mu_0,\ \eta_0^2),\ \tau^2 \sim IG(p, q)$$

Volume: 41, Article ID: e2019013

https://doi.org/10.4178/epih.e2019013

따라서 Random effect model에서는 θ_i에서 θ_k까지 초사전 분포에 의해 각 연구의 치료효과가 연결되므로 더 이상 독립이 아닌 계층적 모형이 된다 [5].

4) 베이지안 방법의 전체 요약

Figure 2를 보면서 간략히 베이지안 방법을 정리해보자.
첫째, 사전분포(사전 확률)을 선정하고 – 공액 사전분포라면 일반적으로 정규분포, 베타분포, 그리고 역감마분포를 주로 활용한다.
둘째, 현재의 자료에서 우도(likelihood)를 계산한 후 베이지안 계층모형을 만든다 – 네트워크 메타분석에서 우도는 치료효과 θ로 주로 표현한다.
셋째, 사전분포와 우도를 MCMC 시뮬레이션에 넣어서 사후분포를 가장 잘 수렴하는 분포를 설정한다. 이때 MCMC 시뮬레이션을 통하여 stable distribution될 확률을 알아 낼 수 있고 더불어 해당 사후분포 함수아래의 면적을 구할 수 있다.
그리고 마지막으로 구해진 사후분포로서 치료효과에 대한 통계적 추론을 실시한다. 따라서 베이지안 네트워크 메타분석에서는 사후분포가 통계에서 일반적으로 사용되는 표준분포가 아니더라도 이를 분석할 수 있는 것이다.

1.2. 빈도주의 방법

빈도주의 방법은 외적정보와는 무관하며 현재의 주어진 자료(present data, likelihood)내에서 연구가설이 참 일 확률은 (p-value 0.05 또는 95% 신뢰구간을 검정) 이미 특정되어 있어서 단지 이것이 받아들여지거나 기각될지를 유의수준으로 판단한다.

https://doi.org/10.4178/epih.e2019013

$$y_{di}^{AJ} = \delta^{AJ} + \beta_{di}^{AJ} + \omega_{d}^{AJ} + \varepsilon_{di}^{AJ}$$

design by treatment interaction model for inconsistency. A는 참조치료, J는 비교치료(J=B,C,...), d는 연구 디자인, i는 d번째 연구디자인에서 i번째 연구를 나타냄.

design by treatment interaction model은 연구 간 이질성과 연구디자인 간 비일관성을 동시에 고려한 빈도주의 네트워크 메타분석 모형이다 [4].

y_{di}^{AJ} 는 연구 간 이질성과 연구디자인 간 비일관성을 고려하여 d 연구디자인 i번째 연구에서 A 참조중재에(reference treatment) 대비한 J 치료의 효과크기를 나타내며 평균의 차이(mean difference), log risk-ratio, 또는 log OR(odds ratio)등으로 나타내어진다. 이때 δ^{AJ}는 일차 관심지표로서 치료비교(treatment contrast); A 참조중재에 대비한 J 중재의 효과크기를 나타낸다. β_{di}^{AJ}는 d 라는 동일 연구디자인 내에서 연구간 이질성(heterogeneity)를 나타낸다. 일반적인 메타분석에서 random effect model로 연구간 변량인 τ2 산출하는 것과 동일하다. ω_{d}^{AJ} 는 비일관성(nconsistency) 뜻하며 앞서 loop inconsistency는 Lu and Ades를 기초로 하였고 design inconsistency는 연구디자인별 차이를 메타회귀분석을 통하여 접근하였다. 물론 consistency 가정을 충족하는 consistency model에서는 모든 연구디자인과 비교중재의 $\omega_{d}^{AJ} = 0$이다.

Volume: 41, Article ID: e2019013

https://doi.org/10.4178/epih.e2019013

R의 "gemtc" 패키지를 이용한 베이지안 네트워크 메타분석

R의 베이지안 방법을 이용한 네트워크 메타분석 패키지인 gemtc를 활용한 순서도를 Figure 3에 나타내었다. 최초 자료 코딩시 해당 함수에 적합하도록 변수명을 고정해야하며 네트워크셋업 -> 네트워크 모델선정(Fixed or Random) -> MCMC 수렴도 최적모델 선정 -> 최종 모델에서 통계적 추론의 순서로 진행한다 (Figure 3).

R에서 네트워크 메타분석을 실시하는 패키지는 베이지안(Bayesian) 네트워크 메타분석을 위한 "gemtc"와 빈도주의(Frequentist) 네트워크 메타분석을 위한 "netmeta"가 있으며 본격적인 분석에 앞서서 해당 패키지를 아래 명령어를 사용하여 설치한다. 보다 상세한 설명은 각 패키지별 상세 코드, 자료, 참고문헌 등이 있으니 참고하기 바란다 [6].

```
·install.packages("nemeta")
·install.packages("gemtc")
```

베이지안 네트워크 메타분석을 실행할 때 Markov Chain Monte Carlo (MCMC) 시뮬레이션을 이용하는데 이에 필요한 프로그램이 JAGS(Just Another Gibbs Sampler, MCMC의 대표적인 방법이 Gibbs Sampler이다)이며 최신버전 4.0 이상을 구글에서 다운받아 설치한다. 또한 설치된 JAGS를 R에서 이용할 수 있게 "rjags" 패키지를 설치한다.

```
·install.packages("rjags")
```

본문과의 구분을 위하여 명령어 앞에는 '·'으로 표시하였다. 명령어가 길어져서 다음 줄로 넘어가더라도 '·' 없으면 앞의 줄에서 이어지는 것이다. 따라서 실제 R 프로그램에 입력시에는 '·'는 제외하고 타이핑하여야 한다.

Volume: 41, Article ID: e2019013

https://doi.org/10.4178/epih.e2019013

1) 데이터 코딩 및 불러오기

베이지안 네트워크 메타분석을 실행하기위해 gemtc 패키지를 로딩시킨다.

·library(gemtc)

다음은 작업폴더에 넣어둔 예제 파일을 아래의 명령어로 R의 메모리에 불러온다. 한가지 주의할 점은 R에서는 쉼표로 구분된 수치파일(csv)의 형태를 선호하니 Supplementary material Table S1을 “bin_dn.csv” 포멧으로 저장해서 지정된 작업폴더에 넣어 두어야한다.

·data_b_bin = read.csv("bin_dn.csv", header=TRUE)

read.csv는 csv파일을 불러오는 함수로서 파일명 “bin_dn.csv"를 불러와서 파일의 첫 번째 변수명을 그대로 쓴다는 뜻이다(header=TRUE). 이렇게 로딩된 파일은 R 메모리에서는 data_b_bin 이름의 데이터로 저장된다. 이를 확인해보려면 View 함수에 지정한 데이터를 넣어준다.

·View(data_b_bin)

gemtc 패키지는 하위에 여러 함수들을 포함하는데 그중 mtc.network 함수는 데이터의 함수명이 특정 변수명이어야만 실행이 가능하다. 이분형 자료에서는 "study", "responders", "sampleSize", "treatment"와 일치해야한다. 본 예제자료는 변수명이 다르므로 colnames 명령어로 변수명을 변경한다.

·colnames(data_b_bin) <- c("study","responders", "sampleSize", "treatment")

colnames는 변수명(열)의 이름을 지정하는데 data_b_bin 데이터의 변수명을 순서대로 변경한다.

2) 네트워크 셋업

Volume: 41, Article ID: e2019013

https://doi.org/10.4178/epih.e2019013

준비된 data_b_bin 데이터를 네트워크 분석을 위해 mtc.network 함수를 사용하여 셋업한다.

```
·network_b_bin <- mtc.network(data.ab=data_b_bin, description="Bayesian NMA binary data")
```

mtc.network 함수는 앞에서 설정된 data_b_bin을 가져다 네트워크 셋업을 시켜 이를 network_b_bin로 선언한다.

```
·plot(network_b_bin)
```

네트워크를 구성하는 치료그룹 간의 직접비교(direct comparison) 관계를 도식화해서 보여준다 (Figure 4). 각 node를 연결하는 엣지(edge)의 굵기는 데이터의 양을 의미한다. Network plot은 네트워크 메타분석 연구에서 치료들 간의 질적·양적관계를 가늠할 수 있기에 필수적으로 제시되어야 한다.

```
·summary(network_b_bin)
```

네트워크 셋업의 전반적인 상황을 볼 수 있다. 각 치료별 개수와 비교군이 2-arm 또는 3-arm 연구의 개수와 개별 치료별 대응개수도 알 수 있다. 앞의 네트워크 plot을 수치적으로 설명한다고 볼 수 있다.

3) 네트워크 모델

네트워크 셋업이 끝났으면 고정효과모형(fixed effect model) 또는 랜덤효과모형(random effect model)의 네트워크 모델을 설정한다.

```
·model_b_bin_fe <- mtc.model(network_b_bin, linearModel='fixed', n.chain = 4)
·model_b_bin_re <- mtc.model(network_b_bin, linearModel='random', n.chain = 4)
```

mtc.model 함수에 네트워크 셋업 데이터 network_b_bin을 불러들여 고정효과모형과 랜덤효과모형을 각각 model_b_bin_fe와 model_b_bin_re로 설정한다. n.chain은

Volume: 41, Article ID: e2019013

https://doi.org/10.4178/epih.e2019013

이어서 진행 할 MCMC 시뮬레이션에서 진행할 chain의 수를 나타낸다.

4) MCMC 시뮬레이션과 수렴여부 진단

(1) MCMC 시뮬레이션 실행

네트워크 모델이 설정되면 MCMC 시뮬레이션을 실시한다. 전반적인 순서는 적정수준의 시뮬레이션 횟수를 설정하여 실행하고 실행한 결과들의 수렴여부를 확인하는 것이다.

우선 고정효과모형을 예제로 상세히 설명하겠다.

```
·mcmc_b_bin_fe <- mtc.run(model_b_bin_fe, n.adapt=5000, n.iter=10000, thin=20)
```

mtc.run 함수에 설정된 고정효과모형 모델을 넣어준다. n.adapt=5000은 시뮬레이션이 시작되는 1-5000번은 버린다는 뜻으로 burn-in이라고 하는데 알고리즘 초기값의 영향을 제거하기 위해 생성된 난수의 초기 일정부분을 제거하는 것이다. n.iter=10000는 10000번 반복 시뮬레이션 실시하며 thin = 20은 20번째 간격으로 추출한다는 의미이다.

다시한번 정리해서 설명하면 시뮬레이션을 실행하는데 있어서 초기값의 영향을 줄이기 위해 1번에서 5000번까지는 버리고 5001번부터 15000번까지 10000번 실시하게 된다. 이때 20번째 간격 5020, 5040.... 순서로 추출하게 된다.

베이지안 분석에서는 사후분포(posterior distribution)를 결정하기 위해 다중연쇄를(multi chain) 고려한 사전분포(prior distribution)를 넣어주는데, 사전분포를 계산하기 위한 사전분포의 사전모수 즉, 초모수(hyperparameter) d의 초기값을 다중으로 설정하여 (예를 들어 -1, 0, 1, 2 등 4개) 다중연쇄 시뮬레이션을 실시한다. 따라서 10000번 시뮬레이션 중에서 20번째 간격으로 추출하게 되니 각 체인별로는 500개씩의

https://doi.org/10.4178/epih.e2019013

데이터를 추출하게 된다.

상기 설명에 대한 상세설명은 summary 명령어를 통해서 확인한면 된다.

·summary(mcmc_b_bin_fe)

(2) MCMC 시뮬레이션 수렴여부 진단

MCMC 시뮬레이션이 잘 수렴되었는지 확인하기 위해서 아래의 사항들을 복합적으로 확인한다.

■ MCMC error

몬테카를로 오차는 작을수록 높은 정밀도를 나타내어 수렴이 잘되었다고 판단할 수 있다. 따라서 반복 시뮬레이션 횟수를 많이하여 표본의 수를 충분히 확보하고 초기값의 영향을 제거하기위해 burn-in 과정을 거치며 데이터 추출 간격 thin을 잘 조절하여야 한다.

■ DIC(deviance information criterion)

$DIC = \bar{D} + pD$로 나타내는데 $\bar{D}$는 잔차이탈도(residual deviance)의 합이며 pD는 모수수의 추정치로서 결국 DIC는 모형의 적합도와 모형의 복잡성을 동시에 고려한 수치로서 작을수록 좀 더 나은 모형이라고 할 수 있다.

■ Trace plot과 Density plot

Trace plot(시뮬레이션 수행 결과를 육안으로 보여주는 표)는 특정한 패턴이 없이 각 chain들이 얽혀 있어야 잘 수렴한다고 판단한다. Density plot은 사후분포(사후밀도함수)로서 시뮬레이션 반복 횟수에 따라 모양이 매우 다르다면 잘 수렴하지 않았다는 것을 의미한다.

Figure 5_a는 burn-in을 실행하지 않은 상태에서 전체 반복 실행횟수를 100과 500을 넣은 것이다. 처음 100회(Figure 5_a 좌) 실시할 때는 4개의 체인의 변동이 심하고 균등하지 않지만 500회(Figure 5_a 우) 정도에 이르면 어느정도 특정패턴이 없이 균등 해진다. 따라서 최소한 채널당 500회 이상은 시뮬레이션을 실시하는 것이 바람직하다. r결국 초기값의 영향을 제거하기 위한 burn-in은 0-100회까지는 너무 불균등하여 당연히 버려야 하며 최소 500회는 넘어야한다. 본 예제에서는 알고리즘의 영향을 최소화하기 위해서 5000회까지는 버리기로 판단하였다.

Figure 5_b에서 동일하게 10000회 반복 실행을 할 때 추출 간격 thin을 20으로하면 채널당 500개의 자료가 추출되며 thin을 10으로(Figure 5_c) 입력하면 채널당 1000개가 추출되어 표본의 수가 많아지게 되고 Trace 그림이 더욱 균등한 분포를 보여준다. 또한 사후밀도함수(density) 가 1000개의 표본이 좀 더 정규분포를 근사한다고 볼 수 있어서 이를 선택한다.

최종 선택된 MCMC 시뮬레이션 모형은 burn-in 5000을 실시하고 반복 실행횟수는 10000회, 자료는 10번째 간격으로 추출하여 채널당 1000개의 표본을 추출하는 것으로 결정하였다.

·mcmc_b_bin_fe <- mtc.run(model_b_bin_fe, n.adapt=5000, n.iter=10000, thin=10)

추출간격 thin 20과 비교하여 전체 표본수의 증가에 따라 치료그룹 $\overline{AB}$의 MCMC SE가 0.004에서 0.003수준으로 낮아져 정밀도가 높아졌다. 그러나 DIC는 56.24에서 56.63으로 실질적으로 큰 의미는 없는 것으로 판단된다.

■ Gelman-Rubin 통계량과 plot

·gelman.diag(mcmc_b_bin_fe)
·gelman.plot(mcmc_b_bin_fe)

Volume: 41, Article ID: e2019013

https://doi.org/10.4178/epih.e2019013

gelman.diag 명령어는 겔만-루빈 통계량을 콘솔에 표시하며 gelman.plot은 겔만-루빈 plot을 그려준다. 시뮬레이션 반복 횟수가 커짐에 따라 1에 가깝게 나타나며 변동이 안정화되어야 잘 수렴했다고 할 수 있다.

(3) MCMC 시뮬레이션 최종 모델 선정

MCMC 시뮬레이션은 결국 다중연쇄를 위한 적절한 chain수, 초기값 영향제거(burn-in), 반복횟수(interation), 그리고 추출간격(thin)을 복합적으로 잘 조절하여 가장 잘 수렴하는 모델을 선정하여야 한다.

본 예제의 Fixed effect model에서는 초기값의 영향을 충분히 제거하고 반복횟수와 추출간격을 늘리면서 MCMC error와 DIC 변화가 최소화되어 거의 변동이 없고 다양한 plot들의 모습이 안정적인 4개의 chain, 5000번의 burn-in, 10000번의 반복, 그리고 10개 간격의 추출을 선택하였다.

다만 컴퓨터의 사양에 따라 시간이 많이 걸릴 수 있으니 횟수는 적절히 조절하기 바란다.

■ burn-in 5000, iteration 10000, thin 10

```
·mcmc_b_bin_fe <- mtc.run(model_b_bin_fe, n.adapt=5000, n.iter=10000, thin=10)
·plot(mcmc_b_bin_fe)
·summary(mcmc_b_bin_fe)
·gelman.diag(mcmc_b_bin_fe)
·gelman.plot(mcmc_b_bin_fe)
```

5) 일관성 검정(inconsistency test)

네트워크 메타분석의 가정에서 일관성(consistency) 검정은 네트워크 메타분석 결과의 적용 가능 여부를 판가름하는 매우 중요한 도구이다.

```
·nodesplit_b_bin_fe <- mtc.nodesplit(network_b_bin, linearModel='fixed', n.adapt=5000,
n.iter=10000, thin=10)
```

https://doi.org/10.4178/epih.e2019013

```
·plot(nodesplit_b_bin_fe)
·plot(summary(nodesplit_b_bin_fe))
```

mtc.nodesplit 함수에 네트워크 셋업 데이터를 넣어서 일관성 검정을 위한 고정효과 모형 모델 nodesplit_b_bin_fe을 만든다. 이때 MCMC 시뮬레이션도 같이 시행되어 진다.

치료 간의 변동을 육안으로 쉽게 파악할 수 있으며 모든 개별 치료 간의 일관성 검정 결과를 쉽게 파악할 수 있다. 일관성 검정 결과 치료 E vs D의 *p*-value는 0.043로 일관성 없는 것으로 나타났으나 나머지 모든 치료에서는 통계적 유의차를 보이지 않았다. 그러나 보다 강건한 분석을 위해서는 random effect model을 설정하는 것이 바람직하다.

6) 숲그림(forest plot)

네트워크 메타분석을 통해서 치료그룹별 효과크기를 한눈에 알아볼 수 있도록 도식화해서 비교한다.

```
·forest(relative.effect(mcmc_b_bin_fe, t1="A"), digits=3)
```

forest 함수에 MCMC 시뮬레이션을 통한 최종 모델을 넣으면 참조치료를 A로하는 forest plot을 생성한다 (Figure 6).

Placebo에 대비한 모든 치료별 효과크기(OR, 수혈률)가 낮게 나타났으며 95% 신용구간(Credible interval)이 겹치지 않는 다는 것을 알 수 있다.

특히 병용치료방법(E, combination)의 경우 Placebo뿐만 아니라 정맥1회주사법[B, IV(single)], 정맥2회 주사법[C, IV(double)], 그리고 국소도포법[D, Topical] 등 모든 치료에 대비해서도 유의하게 수혈률이 낮음을 알 수 있다.

Volume: 41, Article ID: e2019013

https://doi.org/10.4178/epih.e2019013

7) 치료간 비교우위 선정(treatment ranking)

네트워크 메타분석의 가장 중요한 기능 중의 하나가 치료 간의 비교우위를 선정할 수 있다는 것이다. 다시 말해 치료별 최우선 순위에서 최하위 순위까지 선택되어질 누적확률을 계산할 수 있다.

```
·ranks_b_bin_fe <- rank.probability(mcmc_b_bin_fe, preferredDirection = -1)
·print(ranks_b_bin_fe)
```

rank.probability 함수에 MCMC 최종 모델을 넣는다. preferredDirection은 '-1' 또는 '1' 중에서 효과크기가 작을수록 우수한 것인지 클수록 우수한 것인지에 따라 방향을 설정한다. 본 사례는 참조치료 대비 효과크기가 작을수록 우수한 것이니 '-1'을 설정하였다.

확률 테이블에서 볼 수 있듯이 E(combination)가 best 치료일 경우는 99.8%, 2순위에서 선택될 치료는 C(IV double)가 68.2%, 다음으로는 B(IV single), D(Topical), A(Placebo)의 순위를 알 수 있다.

R의 "netmeta" 패키지를 이용한 빈도주의 네트워크 메타분석

R의 빈도주의 방법을 이용한 네트워크 메타분석 패키지인 netmeta를 활용한 순서도를 Figure 7에 나타내었다. 최초 자료의 형태에 따라 효과크기 자료형태로 변환하며 해당 함수에 적합하도록 변수명을 고정해야 한다. 효과크기 자료형태 -> 네트워크 모델선정(Fixed or Random) -> 최종 모델에서 통계적 추론의 순서로 진행한다.

1) 데이터 코딩 및 불러오기

Volume: 41, Article ID: e2019013

https://doi.org/10.4178/epih.e2019013

빈도주의 네트워크 메타분석을 실행하기위해 netmeta 패키지를 로딩시킨다.

```
·library(netmeta)
```

다음은 작업폴더에 넣어둔 예제 파일을 아래의 명령어로 R의 메모리에 불러온다 (Supplementary material Table S1).

```
·data_f_bin = read.csv("bin_dn.csv", header=TRUE)
```

netmeta 패키지는 효과크기 자료형태 “studlab”, “TE”, “seTE”, “treat1”, “treat2” 변수명이어야만 실행이 가능하다. 본 예제자료는 원자료 형태이므로 이를 효과크기 자료 형태로 변환시켜서 변수명도 일치시켜야 한다.

```
·data_f_bin <- pairwise(trt, event=d, n=n, studlab=study, data=data_f_bin, sm ="OR" )
```

pairwise 함수에서 첫번째 치료변수인 trt를 넣어주고 나머지는 빈도수(event = d), 표본수(n = n), 연구이름(studlab = study)을 일치시킨다. 마지막으로 자료를 OR(odds ratio) 또는 RR(relative risk) 할 것인지 설정한다.

data_f_bin를 다시 열어보면 “study”, “d”, “n”, “trt”에서 변경된 것을 알 수 있을 것이다.

2) 네트워크 모델

효과크기 자료형태로의 변환이 완료되었으면 고정효과모형(fixed effect model) 또는 랜덤효과모형(random effect model)의 네트워크 모델을 설정한다.

```
·network_f_bin_fe <- netmeta(TE, seTE, treat1, treat2, studlab, data = data_f_bin, sm="OR",
reference="A”, comb.fixed=TRUE, comb.random=FALSE)
·network_f_bin_re <- netmeta(TE, seTE, treat1, treat2, studlab, data = data_f_bin,, sm="OR",
reference="A”, comb.fixed=FALSE, comb.random=TRUE)
```

netmeta 함수에 효과크기(TE), 표준오차(seTE), 치료1(treat1), 치료2(treat2), 연구명(studlab), 그리고 data_f_bin를 불러들여 고정효과모형과 랜덤효과모형을 각각

Volume: 41, Article ID: e2019013

https://doi.org/10.4178/epih.e2019013

network_f_bin_fe와 network_f_bin_re로 네트워크 모델설정한다.

(1) 네트워크 plot

```
·tname_f_bin <- c("Placebo", "IV(single)", "IV(double)", "Topical", "Combination")
·netgraph(network_f_bin_fe, labels=tname_f_bin)
```

네트워크 plot에 치료명을 넣어주기 위해서 tname_f_bin을 치료명으로 설정한다. netgraph 함수에 network_f_bin_fe 또는 network_f_bin_re를 넣어서 네트워크를 구성하는 치료그룹 간의 직접비교(direct comparison) 관계를 도식화해서 보여준다. 각 node를 연결하는 엣지(edge)의 굵기는 데이터의 양을 의미한다. Network plot은 네트워크 메타분석 연구에서 치료들 간의 질적·양적관계를 가늠할 수 있기에 필수적으로 제시되어야 한다.

(2) 네트워크 모델 요약 추정치

네트워크 모델이 설정되면 해당 모델에 대해 summary 명령어로 전반적인 요약을 볼 수 있다.

```
·summary(network_f_bin_fe)
·summary(network_f_bin_re)
```

전체 연구는 21개이며, 치료의 개수는 5개, 짝지은 직접비교는 25개, 연구디자인은 6개 ($\overline{AB}$, $\overline{AC}$, $\overline{AD}$, $\overline{AE}$, $\overline{BDE}$, $\overline{ABE}$) 임을 확인할 수 있다. 또한 참조 변수를 A로 하였으며 효과크기는 OR로 나타내었다.

본 예제에서는 연구간 분산(tau)이 0이어서 두 모델의 효과크기가 동일하다.

3) 일관성 검정(inconsistency test)

네트워크 메타분석의 가정에서 일관성(consistency) 검정은 네트워크 메타분석 결과의 적용 가능 여부를 판가름하는 매우 중요한 도구이다.

(1) 전체 모델별 접근(global approach)

각각의 연구 디자인별 비일관성 모델의 회귀계수를 계산한 후 Wald test로 전체 모델에 대한 회귀계수들의 선형성을 검정하는 것이다. STATA 네트워크 메타분석 모델 [4] 과 동일한 전체 모델별 일관성 검정을 실시한다. Fixed & Random 두 모델 동일한 결과를 나타낸다.

·decomp.design(network_f_bin_re)

전체 모델별 일관성 검정결과 *p*-value 0.9942는 귀무가설인 일관성을 지지하므로 본 네트워크 모델을 받아들이는데 무리가 없다.

(2) 개별 치료별 접근(local approach)

·print(netsplit(network_f_bin_re), digits=3)

netsplit 함수에 네트워크 모델을 넣어서 각 치료별 일관성 검정을 실시한다. dgits=3는 편의상 소수점 3자리까지만 표시하라는 의미이다.

Random effect model로 분석하면 Fixed & Random 두 모델을 한번에 확인할 수 있다. 본 예제에서는 연구간 분산(tau)이 0이어서 두 모델의 차이가 없다.

모든 치료별 비교에서 *p*-value가 통계적으로 유의하지 않아 비일관성을 보이지않으므로 다시한번 일관성 모델을 지지한다.

Volume: 41, Article ID: e2019013

https://doi.org/10.4178/epih.e2019013

4) 숲그림(forest plot)

네트워크 메타분석을 통해서 치료그룹별 효과크기를 한눈에 알아볼 수 있도록 도식화해서 비교한다.

```
·forest(network_f_bin_fe, ref = "A", digits=3,   xlab = "Odds Raio")
·forest(network_f_bin_re, ref = "A", digits=3,   xlab = "Odds Raio")
```

forest 함수에 설정한 네트워크 모델을 넣어주고 참조치료로 “A”를 설정한다. digits는 소수점 이하 자리수를 설정하며 xlab은 x축 설명이다. 참조치료를 변경하려면 ref에 다른 치료를 넣어주면 된다. 본 예제에서는 연구간 분산(tau)이 0이어서 두 모델의 효과크기가 동일하다.

Placebo에 대비한 모든 치료별 효과크기(OR, 수혈률)가 낮게 나타났으며 95% 신뢰구간(Confidential interval)이 겹치지 않는 다는 것을 알 수 있다.

특히 병용치료방법(E, combination)의 OR 0.033(0.006, 0.175)은 Placebo뿐만 아니라 정맥1회주사법[B, IV(single)] OR 0.273(0.186, 0.399), 정맥2회 주사법[C, IV(double)] OR 0.229(0.146, 0.360), 그리고 국소도포법[D, Topical] OR(0.329(0.197, 0.550) 등 모든 치료에 대비해서도 통계적으로 유의하게 수혈률이 낮음을 알 수 있다.

5) 치료간 비교우위 선정(treatment ranking)

네트워크 메타분석의 가장 중요한 기능 중의 하나가 치료 간의 비교우위를 선정할 수 있다는 것이다. 다시 말해 치료별 최우선 순위에서 최하위 순위까지 선택되어질 누적확률을 계산할 수 있다.

```
·ranks_f_bin_fe <- netrank(network_f_bin_fe, small.values="good")
·print(ranks_f_bin_fe, sort=FALSE)
```

Volume: 41, Article ID: e2019013

https://doi.org/10.4178/epih.e2019013

```
·ranks_f_bin_re <- netrank(network_f_bin_re, small.values="good")
·print(ranks_f_bin_re, sort=FALSE)
```

netrank함수에 네트워크 모델을 넣어준다. 효과크기가 작을수록 우수하다면 small.values에 good을 넣어주고 클수록 우수하다면 bad로 설정한다. 본 사례는 참조치료 대비 효과크기가 작을수록 우수한 것이니 good을 설정하였다.

확률 테이블에서 볼 수 있듯이 E(combination)가 best 치료일 경우는 99.38%, 2순위에서 선택될 치료는 C(IV double)가 65.43%, 다음으로는 B(IV single), D(Topical), A(Placebo)의 순위를 나타낸다.

베이지안 vs 빈도주의 방법 그리고 R vs STATA 소프트웨어에따른 네트워크 메타분석 결과 비교

Table 1에 베이지안과 빈도주의 네트워크 메타분석을 정리하였다. STATA에서 사용하는 네트워크 메타분석은 회귀분석에 기초한 Design by treatment interaction model로서 연구간 이질성(heterogeneity)과 연구디자인 간 비일관성(inconsistency)을 동시에 고려한 모형이다 [4]. 반면 R의 "netmeta" 패키지에서는 이를 약간 변형한 electrical network model을 사용한다 [7,8].

STATA 모델은 최종 효과크기를 산출하는 Consistency model에서 연구내 변량과 연구간 변량을 동시에 고려하기 때문에 "netmeta"의 Random effect model과 동일한 방법이라고 할 수 있다.

빈도주의 방법에서는 연구간 분산(tau)이 0이어서 Random 과 Fixed effect model은 동일한 값을 나타내며 결국 STATA와 동일한 값이다. 마찬가지로 베이지안 방법에서도 Fixed와 Random effect model는 유사한 값을 나타내며 빈도주의 방법과 거의 일치한 결과를 보인다.

Volume: 41, Article ID: e2019013

https://doi.org/10.4178/epih.e2019013

맺음말

본 연구는 통계학을 전공하지 않은 일반 연구자들도 쉽게 수행할 수 있도록 통계학 이론을 최소화하고 메타분석의 실질적 수행방법에 집중하였다. 즉, 일반 연구자들이 이미 개발되어진 통계방법들을 잘 활용하여 본인의 연구분야에 적합하게 쓰고 해석하기 위함이다.

R의 "gemtc" 패키지를 이용한 베이지안 네트워크 메타분석과 "netmeta"패키지를 이용한 빈도주의 네트워크 메타분석을 비교해보았으며 결국 둘은 동일한 결과를 제시한다는 것을 알 수 있다. 예제로 제시한 이분형 자료이외에 연속형 자료에 대한 상세한 설명은 참고문헌을 참조하기 바란다 [2].
본 연구를 통하여 많은 국내 연구자들이 보다 쉽게 메타분석을 수행하고 관련 연구가 활성화되기를 바란다.

Volume: 41, Article ID: e2019013

https://doi.org/10.4178/epih.e2019013

References

1. Hwang SD & Shim SR. Meta-analysisp; from forest plot to network meta-analysis. Seoul: Hannarae publishing co.; 2018 (Korean). ISBN: 9788955662214

2. Shim SR. Network meta-analysis using R software; Bayesian & Frequentist approach: SDB Lab; 2019 (Korean). ISBN 979-11-965933-1-5.

3. Shim SR, Yoon BH, Shin IS, Bae JM. Network meta-analysis: application and practice using Stata. Epidemiol Health. 2017 Oct 27;39:e2017047.

4. White, I. R. Network meta-analysis. The Stata Journal. 2015;15(4): 951-985.

5. Kim DH, Jang EJ, Hwang JS. Meta-analysis using R & WinBUGS. Free academy; 2014 (Korean). ISBN 978-89-7338-581-2.

6. R software "gemtc" & "netmeta" packages. Available from: https://github.com/gertvv/gemtc or https://github.com/guido-s/netmeta or http://meta-analysis-with-r.org or http://meta-analysis-with-r.org/

7. Rucker, G. Network meta-analysis, electrical networks and graph theory. Research Synthesis Methods. 2012; 3(4): 312–324.

8. Rucker, G. and Schwarzer, G. Reduce dimension or reduce weights? Comparing two approaches to multi-arm studies in network meta-analysis. Statistics in Medicine. 2014; 33:4353–4369.

Volume: 41, Article ID: e2019013

https://doi.org/10.4178/epih.e2019013

Figure legends

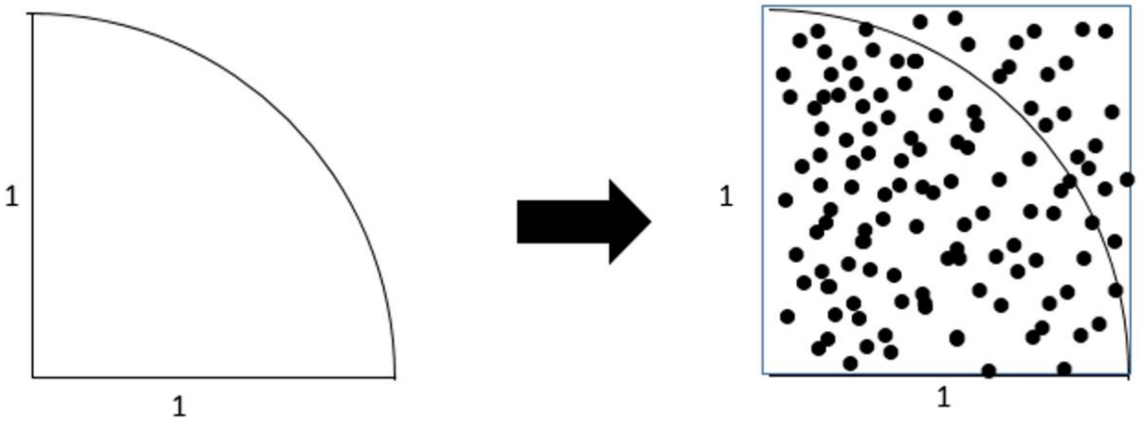

Figure 1. Monte carlo simulation.

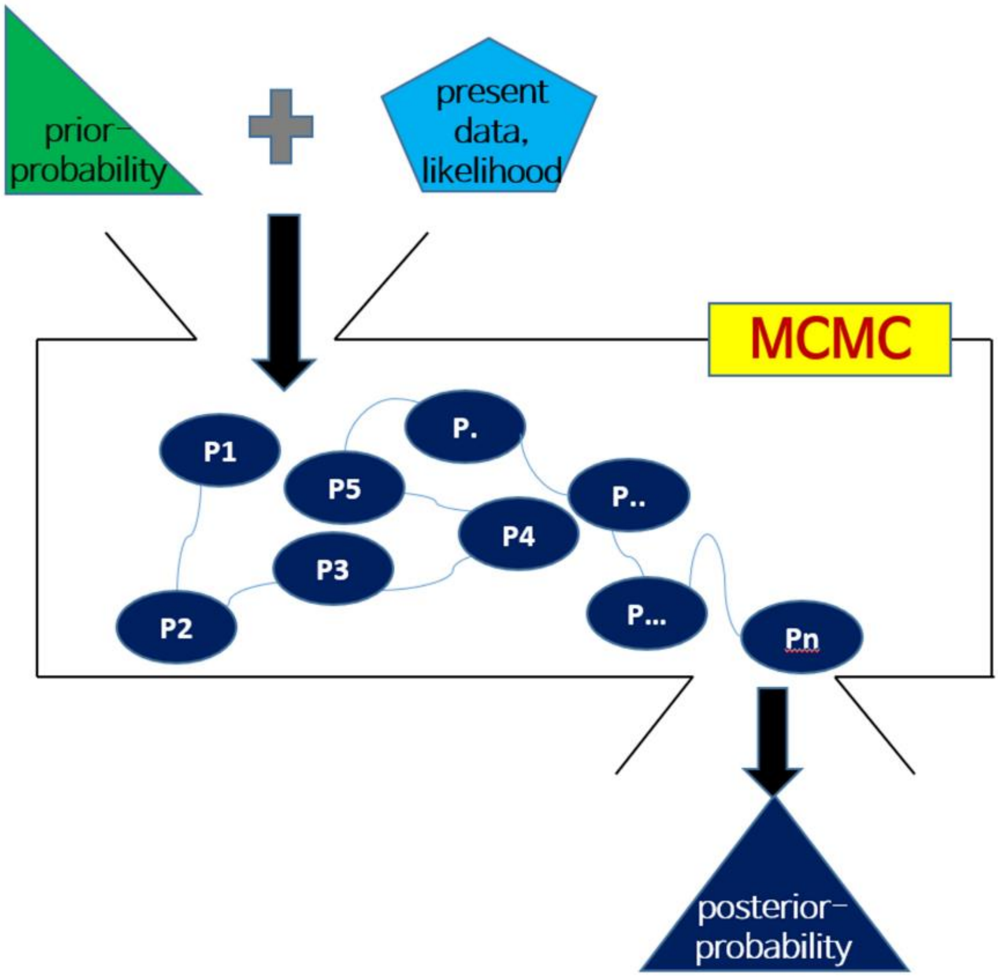

Figure 2. Overall comcept of Bayesian approach using Markov Chain Monte Carlo simulation.

Volume: 41, Article ID: e2019013

https://doi.org/10.4178/epih.e2019013

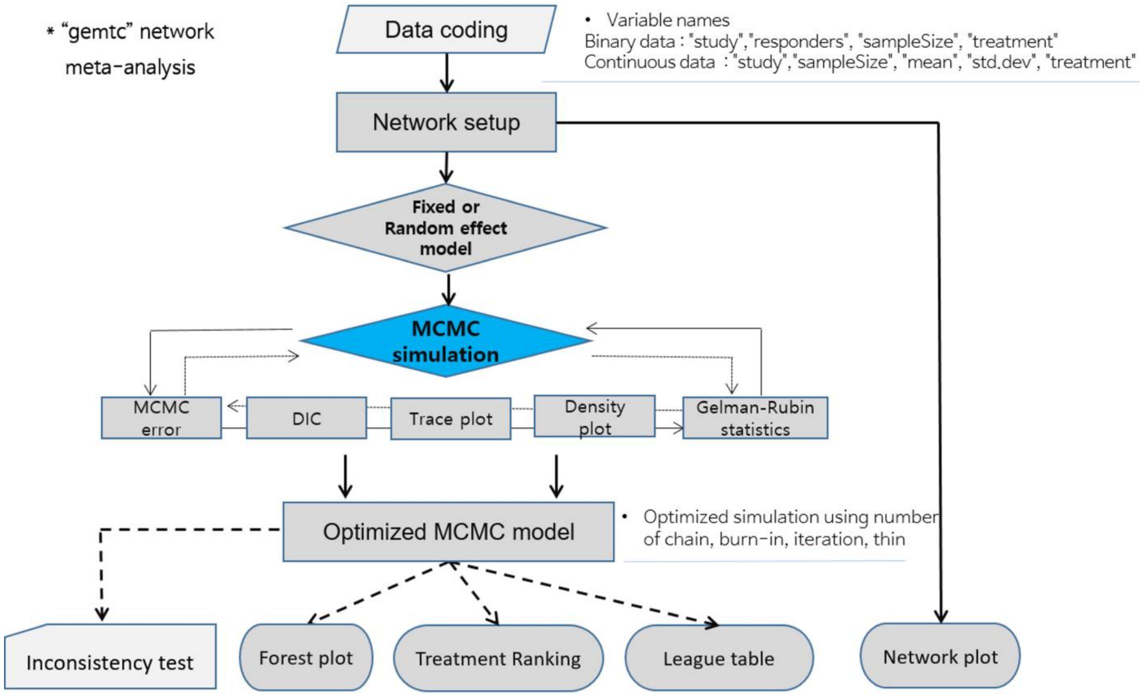

Figure 3. Flow chart of network meta-analysis using R "gemtc" package.

Volume: 41, Article ID: e2019013

https://doi.org/10.4178/epih.e2019013

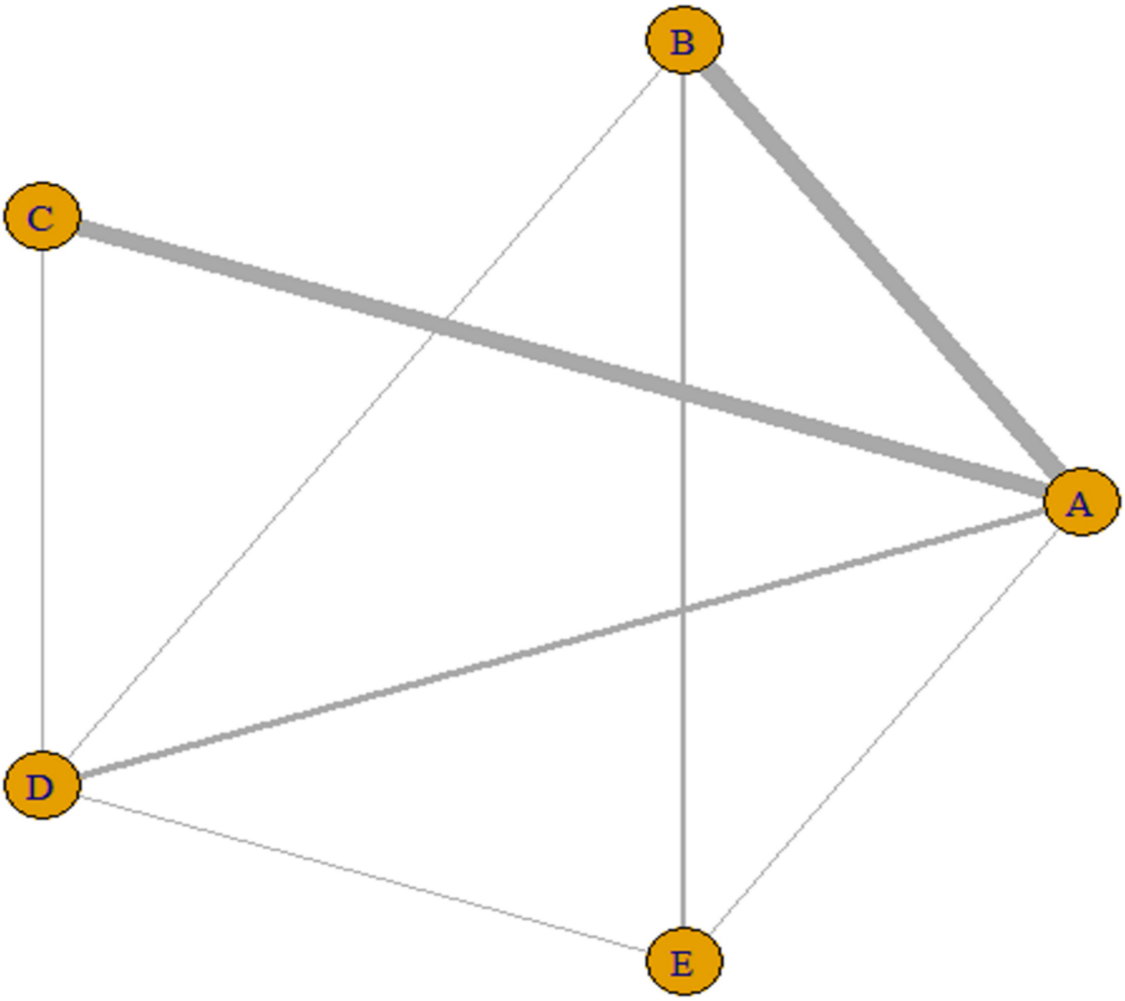

Figure 4. Network plot using "gemtc" package. A, Placebo; B, IV(single); C, IV(double); D, Topical; E, Combination.

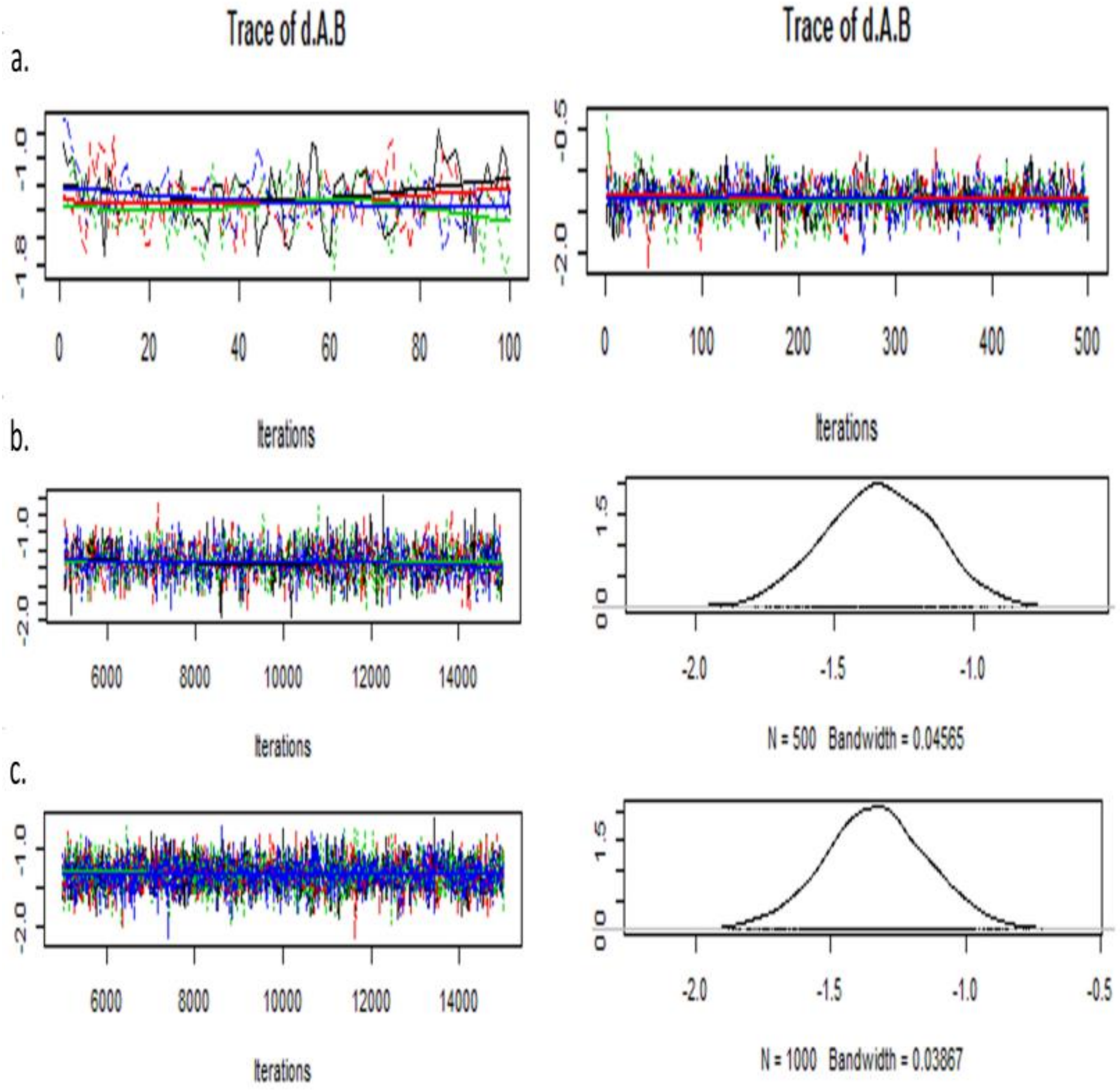

Figure 5. Trace plot and density plot. a. iterations 100 (left) vs 500 (right). b. iterations 10000 & thin 20. c. iterations 10000 & thin 10.

Volume: 41, Article ID: e2019013

https://doi.org/10.4178/epih.e2019013

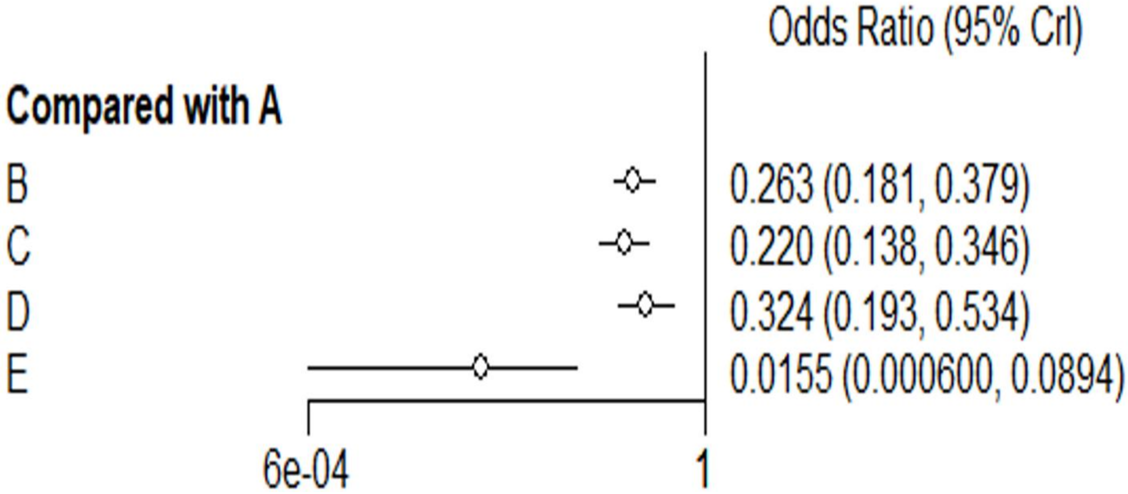

Figure 6. Forest plot_reference A.

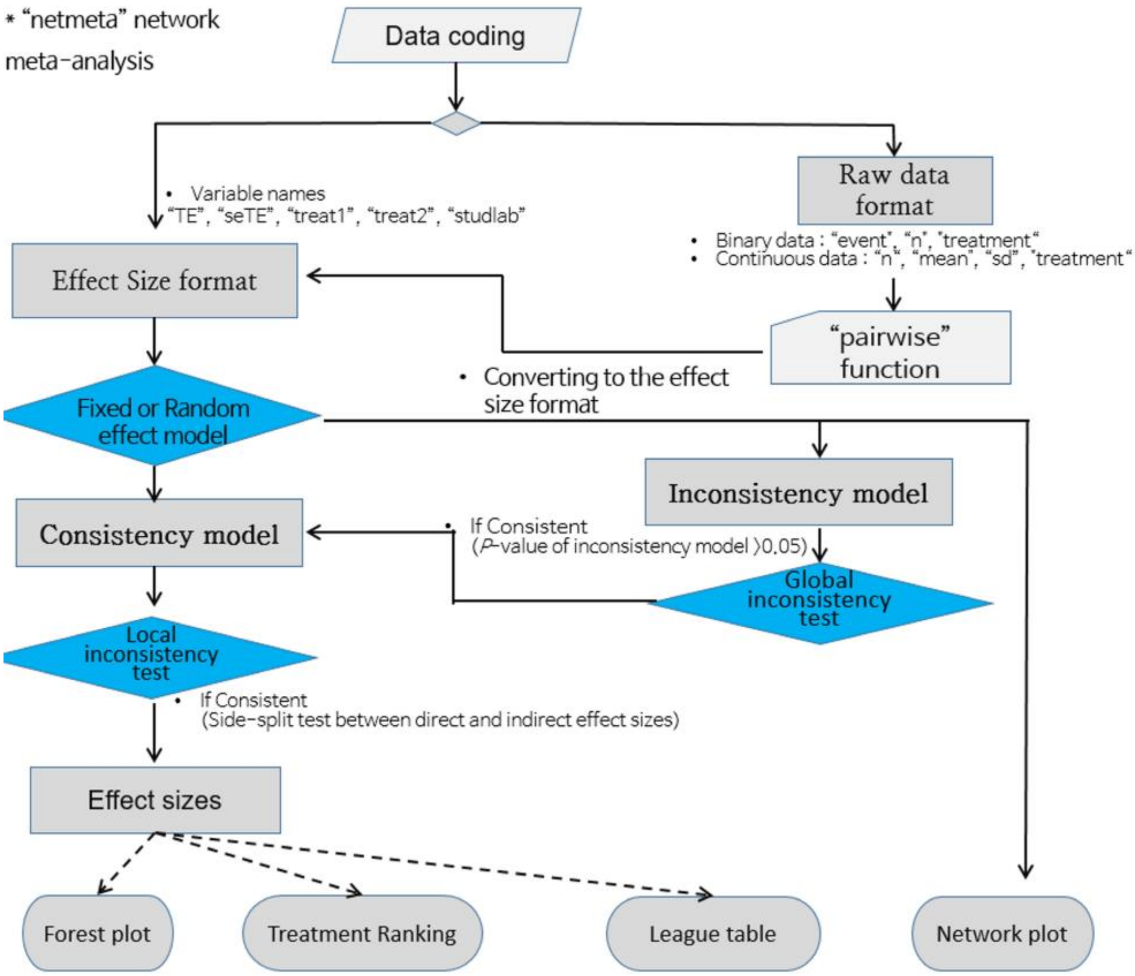

Figure 7. Flow chart of network meta-analysis using R "netmeta" package.
